# Variant-specific *SF3B1* mutations drive distinct splicing and mitochondrial dysfunction in myelodysplastic neoplasms

**DOI:** 10.1186/s40246-026-01000-2

**Published:** 2026-06-18

**Authors:** Andrea Hruštincová, Iva Trsová, David Kundrát, Monika Beličková, Monika Kaisrlíková, Jitka Veselá, Zdeněk Krejčík, Alžběta Hlaváčková, Jiří Suttnar, Hana Štufková, Nikol Volfová, Hana Hansíková, Tatiana Aghová, Zuzana Zemanová, Anna Jonášová, Jaroslav Čermák, Markéta Šťastná Marková, Michaela Dostálová Merkerová

**Affiliations:** 1 Institute of Hematology and Blood Transfusion, Prague, Czech Republic; 2 Department of Genetics and Microbiology, Faculty of Science, Charles University, Prague, Czech Republic; 3 Department of Pediatrics and Inherited Metabolic Disorders, First Faculty of Medicine, Charles University and General University Hospital in Prague, Prague, Czech Republic; 4 Center of Oncocytogenomics, Institute of Medical Biochemistry and Laboratory Diagnostics, First Faculty of Medicine, Charles University and General University Hospital in Prague, Prague, Czech Republic; 5 First Department of Medicine, Department of Hematology, First Faculty of Medicine, Charles University and General University Hospital in Prague, Prague, Czech Republic

**Keywords:** Myelodysplastic neoplasms, *SF3B1*, RNA sequencing

## Abstract

**Background:**

*SF3B1* spliceosome mutations are among the most common genetic lesions in myelodysplastic neoplasms (MDS). The canonical hotspot K700E variant is generally associated with a favorable prognosis, whereas the impact of other recurrent variants is poorly defined. We investigated the clinical, transcriptomic, and functional consequences of distinct *SF3B1* mutations in MDS patients and isogenic cell models.

**Methods:**

Clinical data from 121 *SF3B1*-mutated MDS patients were analyzed. RNA sequencing was performed on CD34⁺ patient bone marrow cells and CRISPR-engineered NALM-6 lines harboring individual *SF3B1* variants. Comprehensive profiling of splicing, gene expression, and mitochondrial bioenergetic parameters was performed.

**Results:**

Compared with K700E, the K666 variant was associated with shorter progression-free survival, distinct splicing abnormalities, and a higher frequency of retained introns, showing partial overlap with the H662 variant. Mitochondria-related genes were frequently mis-spliced and differentially expressed in K666-variant cells. In isogenic models, reduced complex IV activity and marked OXPHOS impairment were observed, with the most pronounced effects seen in K666N. Overall, *SF3B1* variants confer heterogeneous biological and clinical effects. The K666N variant, in particular, is associated with adverse prognosis and profound bioenergetic dysfunction.

**Conclusions:**

These results support a refined view of *SF3B1*-mutated MDS as a biologically heterogeneous entity and suggest that variant-specific mitochondrial vulnerabilities may represent exploitable targets for precision-based therapeutic strategies.

**Supplementary Information:**

The online version contains supplementary material available at https://doi.org/10.1186/s40246-026-01000-2.

## Introduction

Myelodysplastic neoplasms (MDS) constitute a heterogeneous group of hematopoietic stem cell (HSC) disorders that are characterized by bone marrow (BM) dysplasia, ineffective hematopoiesis, and an increased risk of progression to acute myeloid leukemia (AML) [1]. MDS arises through clonal evolution driven by the accumulation of genetic alterations, including chromosomal abnormalities and somatic mutations in a broad range of genes [2, 3].

Somatic mutations in splicing factor genes (*SF3B1*,* SRSF2*,* U2AF1*, and *ZRSR2*) are among the most frequent alterations in MDS, affecting approximately half of MDS patients. *SF3B1* mutations are the most prevalent somatic mutations (~ 30% of patients) [4] and have been shown to be associated with distinct clinical phenotypes, therapeutic responses, and prognoses [5]. *SF3B1* mutations are strongly linked to the presence of ring sideroblasts (RSs), which are abnormal erythroid precursors that are characterized by perinuclear accumulation of iron in the mitochondria [6]. Unlike most MDS-associated mutations, *SF3B1* mutations generally confer favorable outcomes and a lower risk of progression to AML [4]. Consequently, in 2022, the World Health Organization (WHO) designated MDS with mutated *SF3B1* (MDS-SF3B1) as a distinct disease subtype [7].

*SF3B1* encodes a core component of the U2 small nuclear ribonucleoprotein (snRNP), which binds to the pre-mRNA branch point to facilitate spliceosome assembly and activation [8]. In *SF3B1*-mutated cells, alternative 3′ splice site (A3SS) events can generate frameshifted transcripts that may be subject to nonsense-mediated decay (NMD), although the extent of transcript degradation is context-dependent and varies between transcripts and cellular states [9]. Notably, several genes involved in heme biosynthesis and mitochondrial iron metabolism, including *ABCB7*, *PPOX*, and *TMEM14C*, are recurrent targets of *SF3B1*-associated aberrant splicing and have been functionally linked to reduced expression and erythroid phenotypes in SF3B1-mutated myelodysplasia [9]. Enforced expression of *ABCB7* restores erythroid colony formation and reduces mitochondrial ferritin levels, thereby implicating *ABCB7* deficiency as a potential pathogenic driver [10, 11].

Hotspot *SF3B1* variants cluster in the C-terminal HEAT domain [12]. The most common variant, K700E, accounts for approximately half of the *SF3B1* mutations, with other recurrent hotspots at codons 666, 662, and 622. Emerging evidence suggests that specific hotspots—and their co-mutational contexts—may differentially influence the MDS phenotype and prognosis [13, 14]. Only K700E has been consistently associated with improved survival [14], whereas mutations such as K666N have been linked to more aggressive disease courses [13]. However, the functional and prognostic implications of specific *SF3B1* variants have yet to be fully elucidated.

This study aimed to investigate the clinical, transcriptomic, and mitochondrial bioenergetic consequences of distinct *SF3B1* hotspot variants in MDS. By combining patient genomic and transcriptomic data with mechanistic studies in isogenic CRISPR-engineered NALM-6 cell lines, we dissected the functional heterogeneity among the variants. Our results indicate that *SF3B1* variants differ in their clinical impact, splicing and expression profiles, and are associated with variant-specific disturbances in mitochondrial function. These findings reveal previously underappreciated diversity among *SF3B1* variants and confirm that individual variants have distinct pathogenic and prognostic implications in MDS.

## Materials and methods

### Samples

We analyzed 121 patients with *SF3B1*-mutated MDS and 32 mutation-negative controls from two Prague centers. Diagnoses were classified according to the 2022 WHO criteria [7] and risk was assessed via the International Prognostic Scoring System (IPSS-M) [15]. Informed consent was obtained in accordance with the Declaration of Helsinki, and the study was approved by the institutional ethics committee.

RNA sequencing (RNA-seq) was performed on a subset of this clinical cohort selected based on the availability of sufficient material for CD34⁺ cell enrichment, RNA quality suitable for library preparation, and complete clinical and mutational annotation. In total, RNA-seq was generated for 88 MDS samples (56 *SF3B1*-mutated and 32 mutation-negative cases) and 13 healthy donor controls.

Isogenic NALM-6 cell lines harboring wild-type *SF3B1* or CRISPR/Cas9-engineered *SF3B1* variants (K700E, K666N, and H662Q) were used to model variant-specific effects. The edited cell lines were obtained from Horizon Discovery, details of genome editing and validation are provided in the Supplementary Methods. For each genotype, a single independently engineered cell line was analyzed; functional assays were performed in three biological replicates, representing independent experiments conducted on separate culture passages.

### Next-generation sequencing

Targeted mutational profiling was performed using validated myeloid gene panels. Libraries were sequenced on an Illumina MiSeq platform. Variants with a minimum coverage of 300× and variant allele frequency (VAF) ≥ 2% were retained for downstream analyses. Variant annotation was performed using established public databases within an in-house bioinformatics pipeline.

RNA-seq was carried out from ribo-depleted RNA isolated from CD34 + BM cells, and the libraries were sequenced as paired-end reads (2 × 100 bp) on an Illumina NovaSeq platform. Alternative splicing was analyzed using rMATS, considering skipped exons (SE), retained introns (RI), alternative 5′ and 3′ splice sites (A5SS and A3SS), and mutually exclusive exons (MXE). Splicing inclusion levels were quantified as percent spliced-in (PSI), and differences between groups were expressed as ΔPSI. Splicing events with |ΔPSI| > 0.1 and false discovery rate (FDR) < 0.1 were considered significant. Differential gene expression analysis was performed using edgeR, requiring transcripts to have ≥ 5 counts per million (CPM) in at least 50% of samples and an FDR < 0.05. Functional enrichment was evaluated via the DAVID tool [16] and gene set enrichment analysis (GSEA) [17].

### Mitochondrial and metabolic assays

Mitochondrial DNA content, abundance of OXPHOS complexes and subunits, respiratory chain enzyme activities, oxygen consumption rates measured by Seahorse extracellular flux analysis, and targeted metabolite profiling by LC/MS were analyzed using established protocols.

### Statistical analysis

Statistical analyses were performed using GraphPad Prism. Group comparisons were conducted using Student’s t-test. Progression-free survival (PFS) was analyzed using Kaplan–Meier estimates and compared with the log-rank test. Multivariable survival analysis was not performed because of the limited size of the patient subgroups, which would preclude reliable estimation. A p-value < 0.05 was considered statistically significant.

Additional methodological details are provided in the Supplementary Methods.

## Results

### Clinical characterization of *SF3B1*-mutated MDS patients

We analyzed the clinical and molecular features of 121 MDS patients with *SF3B1* mutations. According to the WHO criteria [7], 74 patients fulfilled the definition of MDS with low blasts and an *SF3B1* mutation (MDS-SF3B1), whereas the remaining 47 patients were classified as other MDS subtypes due to additional cytogenetic or molecular abnormalities, and/or increased blast counts. For comparison, 32 MDS patients without detectable mutations in the analyzed gene panel were included as a mutation-negative (wild-type, wt) control group. One *SF3B1*-wild-type patient with a high RS proportion was classified as MDS-LB; in the absence of an *SF3B1* mutation, this patient remained within the wt cohort. The overall clinical and genetic characteristics of the cohort are summarized in Supplementary Tab. S1.

Across the entire cohort of 121 *SF3B1*-mutated patients, the most frequent co-mutations involved *TET2*,* DNMT3A*,* RUNX1*, and *ASXL1*, and 11 patients additionally harbored co-mutations in other splicing factor genes (*SRSF2*,* U2AF1*, or *ZRSR2*). Cytogenetically, most patients had normal karyotypes or isolated del(5q). A detailed breakdown of recurrent co-mutations and major cytogenetic abnormalities across relevant patient subgroups, including K700E and pooled K666-variant cases, is provided in Supplementary Table 2.

Patients stratified by the IPSS-M [15] displayed the expected PFS distribution; however, the moderately high-risk group showed unexpectedly favorable survival (Supplementary Fig. S1A–B). This observation is likely influenced by the study design, which preferentially included patients with *SF3B1* mutations, thereby enriching this risk category for cases with less aggressive disease biology and shifting the expected survival distribution, particularly in the context of a small subgroup (*n* = 15).

We also defined molecular subgroups in accordance with Bernard et al. [15]: SF3B1^5q^ (*SF3B1* mutation with isolated del(5q); *n* = 10), SF3B1^β^ (*SF3B1* mutation with co-mutations in *BCOR*,* BCORL1*,* NRAS*,* RUNX1*,* SRSF2*, or *STAG2*; *n* = 28), and SF3B1^α^ (*SF3B1* mutation with other co-mutations or alone; *n* = 83). As reported, SF3B1^α^ was associated with favorable outcome whereas SF3B1^β^ had poor outcome; interestingly, *SF3B1*^5q^ showed improved survival (Supplementary Fig. S1C), likely due to its small sample size.

### The K666N *SF3B1* variant is associated with poor prognosis

To evaluate the prognostic relevance of individual *SF3B1* variants, we analyzed survival in the full clinical cohort of 121 patients with *SF3B1*-mutated MDS. For the purpose of survival analyses, patients were grouped into four categories: K700E (*n* = 65), K666-variant (any substitution at codon 666; *n* = 20), H662-variant (*n* = 11), and an “other *SF3B1* variants” group comprising the remaining less frequent non-K700E and non-K666 substitutions (*n* = 32).

**Fig. 1:**
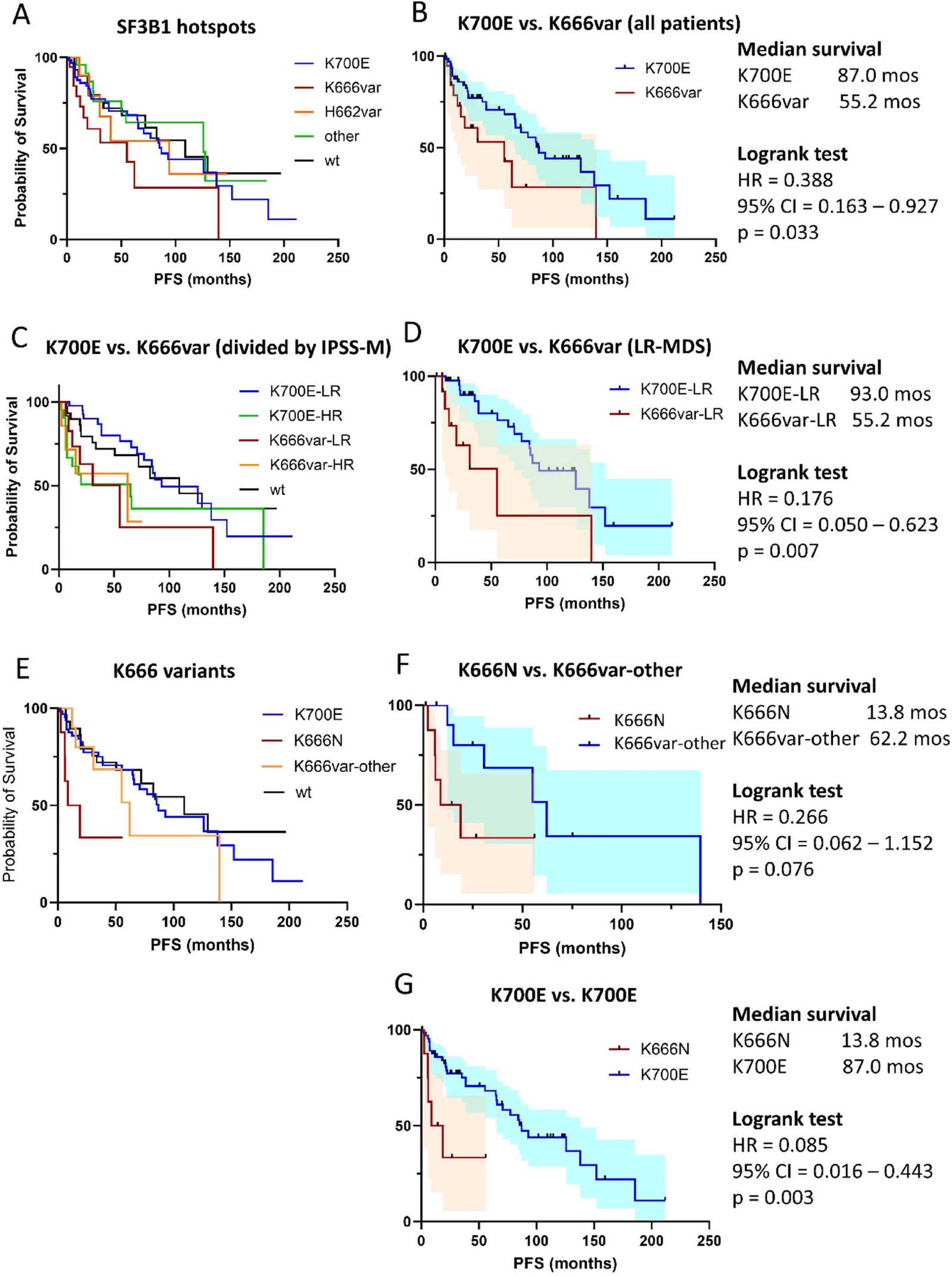
Progression free survival (PFS) of MDS patients with distinct *SF3B1* variants. (A) Kaplan–Meier plots for patients with *SF3B1* mutations at codons K700E, K666-variant (K666var), H662-vatiant (H662var), or other residues, compared with the wild-type group (wt). (B) PFS analysis with the log-rank test for patients with *SF3B1* K700E (*n* = 65) vs. K666-variant (*n* = 20). (C) Kaplan–Meier plots for patients with K700E or K666-variant variants stratified by IPSS-M risk (lower-risk, LR vs. higher-risk, HR). (D) PFS analysis with the log-rank test in lower-risk MDS patients with K700E (*n* = 44) vs. K666-variant (*n* = 13) variants. (E) Kaplan–Meier plots for patients with K666-variants, subdivided into K666N (*n* = 8) and other K666-variants (K666var-other, *n* = 12), along with K700E (*n* = 44) and wt patients. (F) PFS analysis with the log-rank test for K666N (*n* = 8) vs. other K666-variants (*n* = 12) variants. (G) PFS analysis with the log-rank test for K666N (*n* = 8) vs. K700E (*n* = 65) variants

Kaplan–Meier analysis revealed significantly shorter PFS for K666-variant compared to K700E (median PFS: 55.2 vs. 87.0 months, *p* = 0.033; Fig. 1A–B). Although the difference was most pronounced in lower-risk MDS (median PFS: 93 vs. 55.2 months, *p* = 0.0071; Fig. 1C–D), K666-variant conferred poor outcomes across all risk groups, suggesting an intrinsically adverse mutation effect.

Notably, among patients with K666 variants (Kaplan–Meier plot shown in Fig. 1E), those with K666N showed a trend toward shorter PFS compared with patients carrying other K666 variants (13.8 vs. 62.2 months, *p* = 0.077; Fig. 1F) and significantly shorter PFS compared with patients harboring the K700E mutation (13.8 vs. 87.0 months, *p* = 0.003; Fig. 1G). Together, these data suggest that, despite the generally favorable prognosis associated with *SF3B1* mutations, the K666N variant may represent a potentially adverse exception. This interpretation is limited by the small size of the K666N subgroup but is consistent with previous reports describing distinct splicing patterns and less favorable outcomes associated with the K666N variant [13].

### K700E and K666-variant *SF3B1* mutations induce distinct splicing alterations in patient samples

**Fig. 2:**
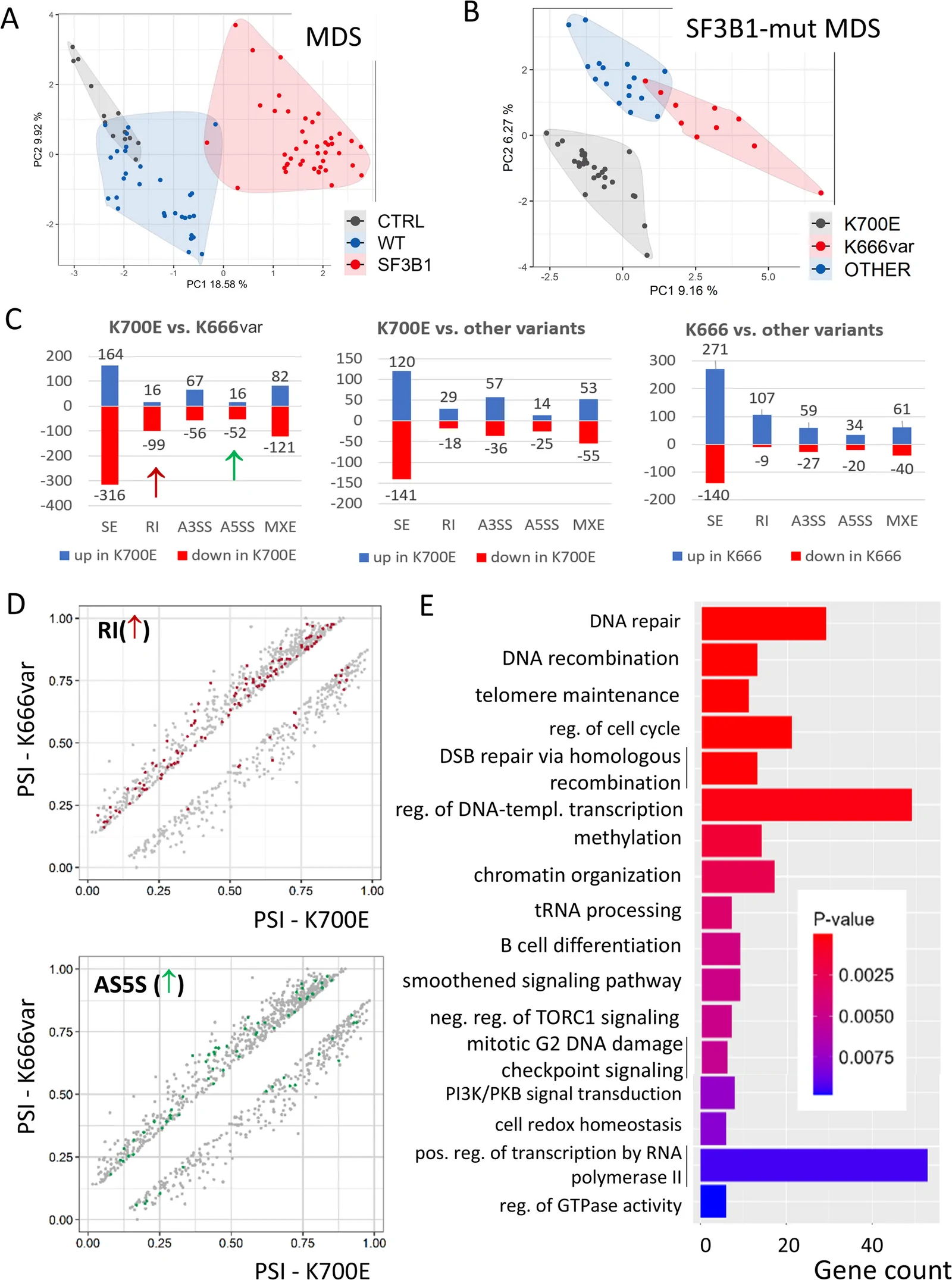
Variant-specific splicing profiles in *SF3B1*-mutated MDS patients. (A) Principal component analysis (PCA) based on splicing profiles in *SF3B1*-mutated MDS patients (SF3B1), wild-type MDS patients (wt), and healthy controls (ctrl). (B) PCA within the *SF3B1*-mutated cohort stratified by hotspot variant (K700E, K666-variant, other). (C) Bar plots showing the number of differentially spliced events (ΔPSI > 0.1, FDR < 0.1) between the indicated *SF3B1* variant groups. The event types include skipped exons (SE), retained introns (RI), alternative 3′ and 5′ splice sites (A3SS and A5SS), and mutually exclusive exons (MXE). (D) Scatter plots of PSI for RI and A5SS events, showing representative changes between K700E and K666-variant. These event types were selected for their large differences in (C) (highlighted by red and green arrows). (E) Gene Ontology biological process (GO BP) enrichment analysis of alternatively spliced genes between K700E and K666-variant. GO BP terms with *p* < 0.01 are shown. The color indicates statistical significance (p value) and the bar length indicates the number of genes

RNA-seq was performed on CD34 + BM samples from 88 MDS patients (56 with *SF3B1* mutations and 32 without detectable pathogenic mutations in the tested gene panel) and 13 healthy donors. Principal component analysis (PCA) based on splicing profiles separated *SF3B1*-mutated cases from mutation-negative patients and healthy controls (Fig. 2A). Because transcriptomic differences between *SF3B1*-mutated and mutation-negative MDS or healthy controls have been extensively characterized previously [9, 12, 18], subsequent analyses focused specifically on heterogeneity among distinct *SF3B1* variants.

Within the *SF3B1*-mutated cohort, samples carrying the two most frequent hotspot variants, K700E and K666 substitutions, formed distinct clusters in PCA, clearly separated from each other as well as from samples harboring other, less frequent *SF3B1* variants, indicating variant-specific splicing signatures (Fig. 2B). Based on this separation, RNA-seq analyses were designed to compare K700E (*n* = 30) and K666-variant cases (*n* = 10). The K666-variant group comprised K666N (*n* = 3), K666E (*n* = 2), K666R (*n* = 2), K666T (*n* = 2), and K666M (*n* = 1). Owing to the limited number of samples per individual substitution, analyses were performed at the level of the pooled K666-variant group, and results are presented as relative differences between K700E and K666 hotspot variants rather than as absolute deviations from a baseline state. The clinical and molecular characteristics of patients included in these RNA-seq analyses are summarized in Supplementary Tab. S3.

Differential splicing analysis between the K700E versus K666-variant patient samples revealed 1,007 significantly altered splicing events across 711 genes (ΔPSI > 0.1, FDR < 0.1). The most frequent event types were RI, A5SS, and SE, all showing higher inclusion rates in the K666-variant samples (Fig. 2C–D). Among the most strongly affected genes were *HDAC9*,* FOXRED1*, and *RPS15*, with RI ΔPSI values of 0.309, 0.268 and 0.250, respectively. Gene Ontology (GO) analysis revealed significant enrichment of pathways involved in DNA repair and recombination, telomere maintenance, and cell-cycle regulation (*p* < 0.01; Fig. 2E). Prominent mis-spliced genes included *BRCA1*,* POLG*,* RAD50*,* RAD51D*,* NPM1*, and *ATM* (see Supplementary Tab. S4 for the full list).

### K700E and K666-variant *SF3B1* mutations differentially impact gene expression in patient samples

**Fig. 3:**
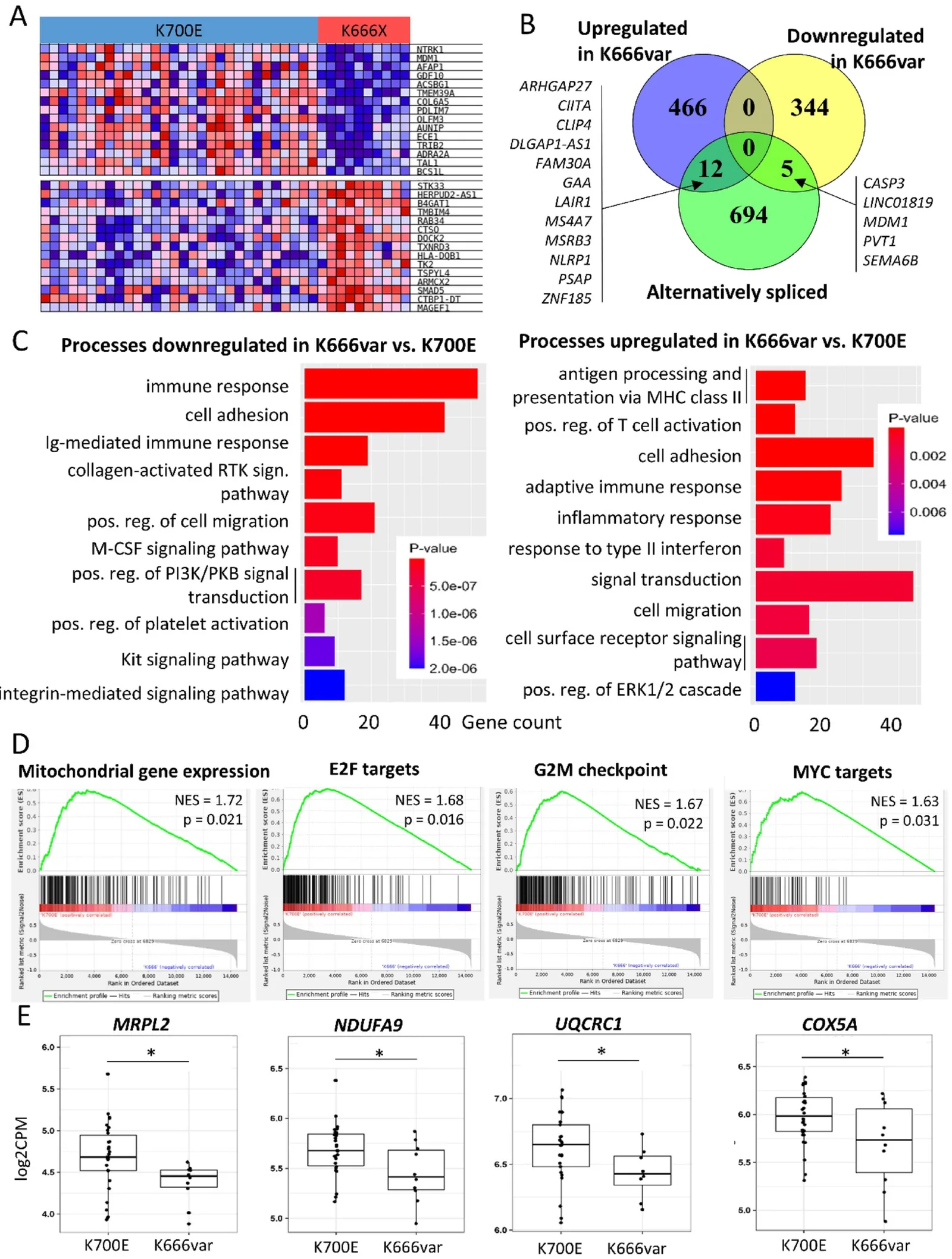
Differential gene expression between MDS patients with K700E and K666-variants. (A) Heatmap of the top 30 differentially expressed genes. Expression values are shown as colors (red, higher expression; blue, lower expression) and reflect relative differences between K700E and K666-variant (K666var) samples, rather than absolute changes relative to mutation-negative or healthy controls. (B) Venn diagram showing overlap between genes with altered splicing (ΔPSI > 0.1, FDR < 0.1) and genes with differential expression (FDR < 0.05). (C) Top 10 enriched GO biological processes from DAVID pathway enrichment analysis. The color indicates statistical significance (p value) and the bar length indicates the number of genes. (D) Gene set enrichment analysis (GSEA) of Hallmark pathways with *p* < 0.05. (E) Boxplots of selected mitochondria-related genes (*MRPL2*,* NDUFA9*,* UQCRC1*, and *COX5A*). The log2CPM values for the K700E and K666-variant samples are shown. *FDR < 0.05

Differential gene expression analysis of the K700E versus K666-variant patient samples revealed 349 genes with lower expression and 478 genes with higher expression in K700E relative to the K666-variant group (FDR < 0.05; Fig. 3A). Only 17 genes overlapped between the 711 differentially spliced genes and the set of differentially expressed genes (Fig. 3B), indicating that the primary impact of *SF3B1* variants in patient CD34⁺ progenitor samples is reflected at the level of isoform usage rather than as widespread changes in gene-level transcript abundance, consistent with context-dependent engagement of NMD.

DAVID pathway analysis revealed variant-specific enrichment of biological processes, particularly immunity, cell adhesion, and migration (Fig. 3C). Compared with K700E, K666-variant samples exhibited relatively lower expression of 50 immunoglobulin-related genes—including *IGHG1*,* IGKC*,* IGHA1*, and multiple variable region genes—suggesting suppression of early B-lineage transcriptional programs. Conversely, K666-variant samples showed relatively higher expression of several HLA genes (*HLA-DRA*,* DPA1*,* DRB1*,* DQA1*, and *HLA-B*), contributing to enrichment of antigen presentation and adaptive immune response pathways. Additionally, IFN-stimulated inflammatory signatures were more prominent in the K666-variant samples.

GSEA further revealed enrichment of genes related to mitochondrial gene expression, E2F targets, G2M checkpoints, and MYC-related pathways (*p* < 0.05, Fig. 3D). Notably, K666-variant samples showed relatively reduced expression of mitochondrial ribosomal subunits (*n* = 46) and oxidative phosphorylation (OXPHOS) components—including *NDUF*,* UQCRC*, and *COX* subunits—compared with K700E samples (Supplementary Fig. S2). The expression levels of representative genes (*MRPL2*,* NDUFA9*,* UQCRC1*, and *COX5A*) are shown in Fig. 3E.

### Isogenic NALM-6 models confirm distinct effects of *SF3B1* variants

To isolate the specific effects of *SF3B1* variants from the complex molecular background of MDS patients, we used isogenic NALM-6 cell lines engineered to carry either the wild-type allele or the K700E, K666N, or H662Q *SF3B1* variants. Across all experiments with isogenic NALM-6 cell lines, analyses were performed using at least three biological replicates, representing independent experiments conducted on separate culture passages of the same clone.

**Fig. 4:**
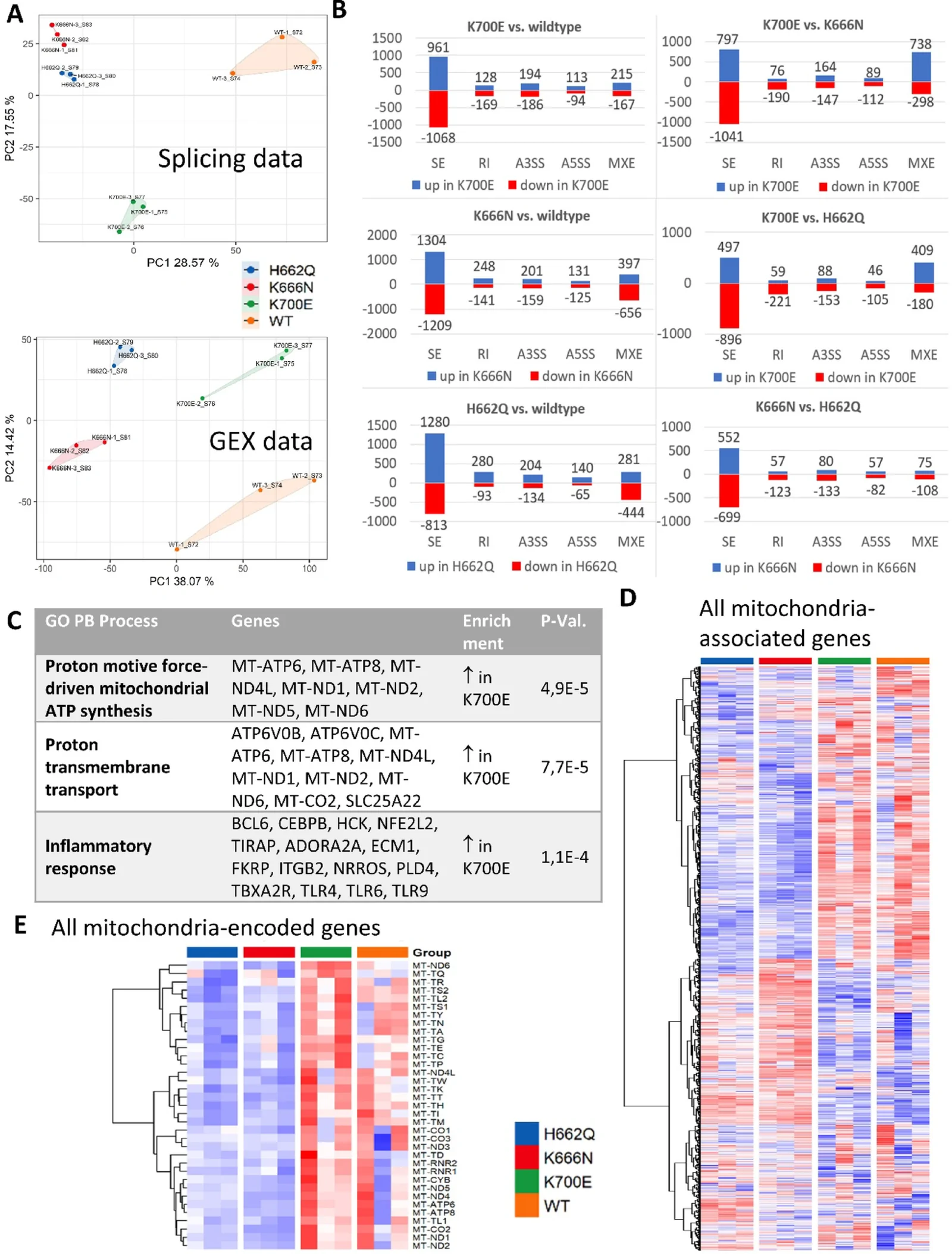
Transcriptomic differences among *SF3B1* variants in isogenic NALM-6 cell models. (A) Principal component analysis (PCA) of splicing and gene expression (GEX) data from four clones (wt, K700E, K666N, H662Q). (B) Boxplots of differentially spliced events (ΔPSI > 0.1, FDR < 0.1) between the clones. SE - skipped exons, ASS5 - alternative 5’ splice sites, ASS3 - alternative 3’ splice sites, MXE - mutually exclusive exons, RI - retained introns. (C) Gene Ontology biological process (GO BP) enrichment of genes differentially expressed between K700E and K666N clones (terms with *p* < 0.01 are shown). (D) Heatmap of the expression of all mitochondria-associated genes in the four clones. The expression values are represented as colors, where the range of colors represents the range of expression values (red, upregulation; blue, downregulation). (E) Heatmap of the expression of all mitochondria-encoded genes in the four clones (color scale as in D)

RNA sequencing of NALM-6 cell lines revealed variant-specific transcriptomic profiles, with clustering analyses clearly separating the lines (Fig. 4A). Compared with patient samples, a greater number of alternative splicing events were detected. The most pronounced difference was again observed in the RI frequency, which was greater in K666N and H662Q cells than in wild-type and K700E cells. Additional differences were noted across other splicing categories: SE predominated in K666N and H662Q cells, ASS5 predominated in H662Q cells, and MXE predominated in K700E and wild-type cells. Overall, the splicing profiles were more similar between the wild-type and K700E lines and between the K666N and H662Q lines (Fig. 4B). The functional annotation of the 516 genes with the most divergent splicing events (ΔPSI > 0.25, FDR < 0.01) between K666N and K700E revealed enrichment of genes related to mitochondrial organization (*AGTPBP1*,* ATP7A*,* BCS1L*,* FANCG*,* LETMD1*,* DNM1L*,* LETM2*,* MSTO1*,* MIEF1*,* MFF*,* PHB2*, and *TERT;* *p* = 4.0 × 10⁻⁵) and metabolic pathways (57 genes, *p* = 5.2 × 10⁻⁵).

To integrate patient and cell line splicing data, we identified 203 mis-spliced events in 180 genes common to K666N vs. K700E in both the patient and NALM-6 comparisons. DAVID annotation revealed that many genes encode transcriptional regulators, including *BRCA1*,* CREBZF*,* HDAC8*,* MAP3K7*,* PVT1*, and *STAT6*. Notably, six of these genes—*COA8*,* COASY*,* FOXRED1*,* METTL8*,* NAPRT*, and *TAFAZZIN*—are essential for mitochondrial homeostasis.

Expression profiling of NALM-6 confirmed that the wild-type and K700E cells were similar, whereas K666N cells resembled H662Q. The greatest expression differences were between K700E and K666N (Supplementary Tab. S5). Consistent with the patient data, only 13 genes overlapped between the differentially spliced (*n* = 996) and differentially expressed (*n* = 476) gene sets (Supplementary Fig. S3), suggesting that splicing changes rarely affect total mRNA levels. Pathway analysis revealed that K666N cells had downregulated mitochondrial proton transport and inflammatory genes (Fig. 4C), including the genes encoding OXPHOS components (e.g., mitochondrially encoded NADH dehydrogenase, ATP synthase, and cytochrome c oxidase subunits) and Toll-like receptors (*TLR4*,* TLR6*, and *TLR9*). Importantly, ANOVA across 1,595 mitochondria-associated genes revealed that 664 genes were differentially expressed (FDR < 0.05), with wild-type and K700E showing similar expression profiles and K666N clustering with H662Q (Fig. 4D). The expression of all mitochondrially encoded genes was markedly suppressed in both the K666N and H662Q lines (Fig. 4E).

### Distinct *SF3B1* variants differentially affect OXPHOS complexes

Given the transcriptomic evidence of mitochondrial alternations between the K666N and K700E variants, we examined OXPHOS integrity in four isogenic NALM-6 clones, which provided sufficient biological material and eliminated interindividual variability inherent to primary samples.

**Fig. 5:**
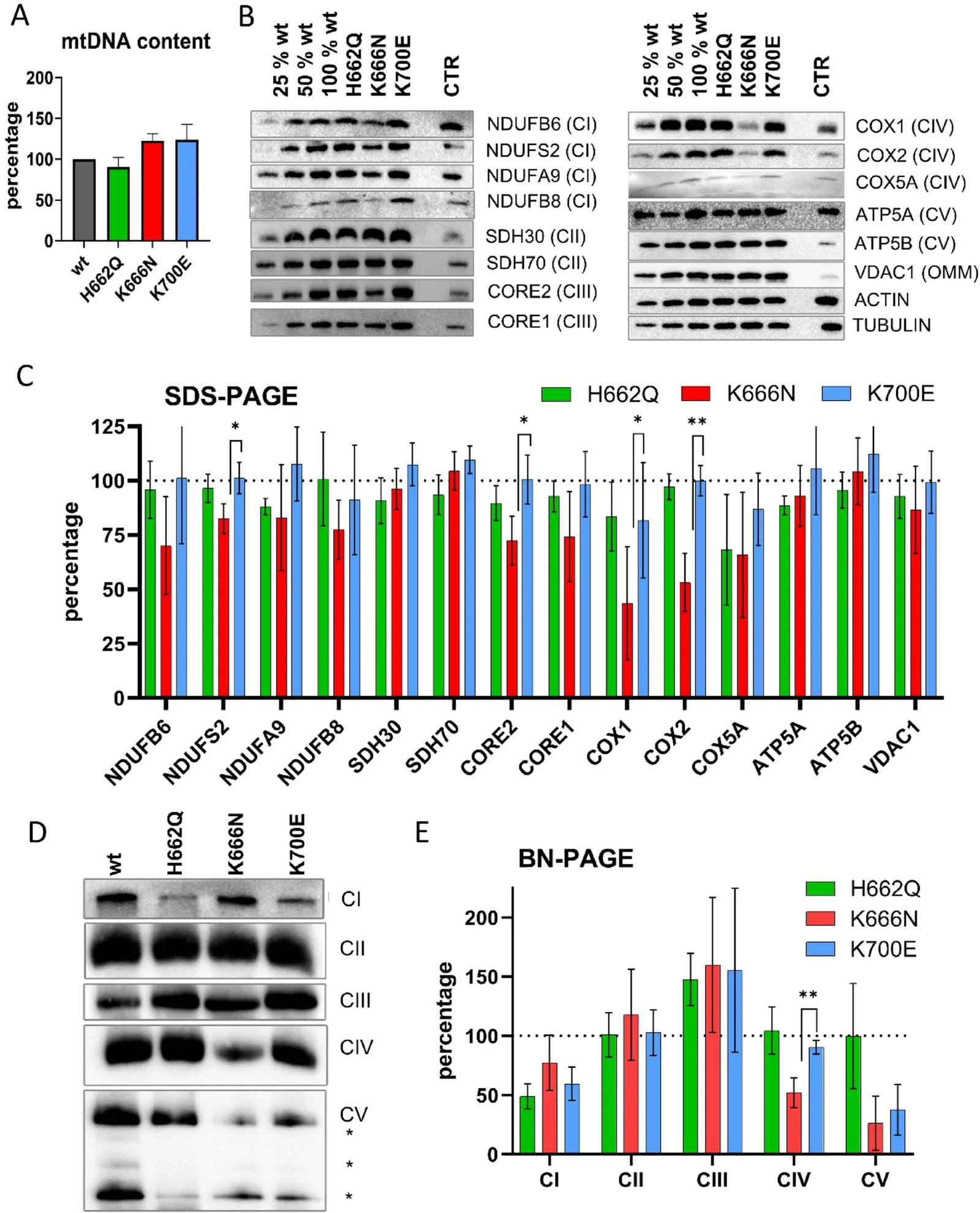
Contents of mitochondrial DNA (mtDNA) and oxidative phosphorylation system (OXPHOS) complexes in isogenic NALM-6 cell models. (A) Relative mtDNA content measured by qPCR for the *MT-RNR2* gene normalized to that of *GAPDH* (mean ± SD, *n* = 3) in cell lines with different *SF3B1* variants (wt, K700E, K666N, H662Q). (B) Representative Western blots (SDS‒PAGE) of selected subunits of OXPHOS complexes I–V. Actin and tubulin are shown as loading controls; VDAC1 is a mitochondrial marker that is localized in the outer mitochondrial membrane (OMM); a fibroblast sample was used as another control (CTR). (C) Quantification of SDS‒PAGE results; the results were normalized to those of actin (mean ± SD, *n* = 3). WT values are indicated by a horizontal reference line at 100%. (D) Western blot analysis of OXPHOS complexes (CI–CV) under native conditions (BN‒PAGE). Complex II was used as an internal loading control. Asterisks indicate ATP synthase (CV) subcomplexes. (E) Quantification of BN‒PAGE results (mean ± SD, *n* = 3). *p < 0.05, **p < 0.01. WT signal intensity is shown as a horizontal reference line at 100%

Mitochondrial DNA quantification revealed only minor differences; mtDNA levels ranged from 90% to 124% of those of wild-type, which was insufficient to explain the marked reduction in the transcription of mitochondrially encoded genes (Fig. 5A).

The abundance of selected OXPHOS subunits was analyzed by SDS‒PAGE and Western blotting. K666N cells presented the greatest reduction, particularly in abundance of complex IV (MT-COX1, MT-COX2, and COX5A reduced to ~ 50% of that of other clones), and to a lesser extent, of complex I (NDUFB6, NDUFS2, NDUFA9, and NDUFB8) and complex III (CORE1 and CORE2) (Fig. 5B–C). Representative full-length, uncropped blots, including composite panels derived from three independent biological replicates, are provided in Supplementary Fig. 4.

Blue Native PAGE (BN‒PAGE) analysis of intact complexes (Fig. 5D–E) confirmed an ~ 50% reduction in complex IV in K666N, with additional alterations in complexes I, III, and V. Representative full-length, uncropped BN-PAGE membranes are shown in Supplementary Fig. 5, including composite panels derived from three independent biological replicates as well as a representative full-length gel from an individual experiment. Complex II remained stable, thus validating its use as a loading control. Notably, complex I assembly was reduced in K700E and H662Q, despite normal or increased subunit levels observed via SDS‒PAGE, highlighting the disconnect between subunit abundance and complex integrity. Complex V also showed clone-specific variation, with lower levels in K666N and K700E, and altered ATP synthase subcomplex distribution. This pattern suggests differences in assembly dynamics or stability, rather than sample degradation, which would be expected to affect all samples similarly. Complex V integrity was best preserved in H662Q.

Together, these findings demonstrate that *SF3B1* variants differentially impair OXPHOS, with K666N causing the most severe disruption.

### K666N *SF3B1* variant impairs mitochondrial bioenergetics

**Fig. 6:**
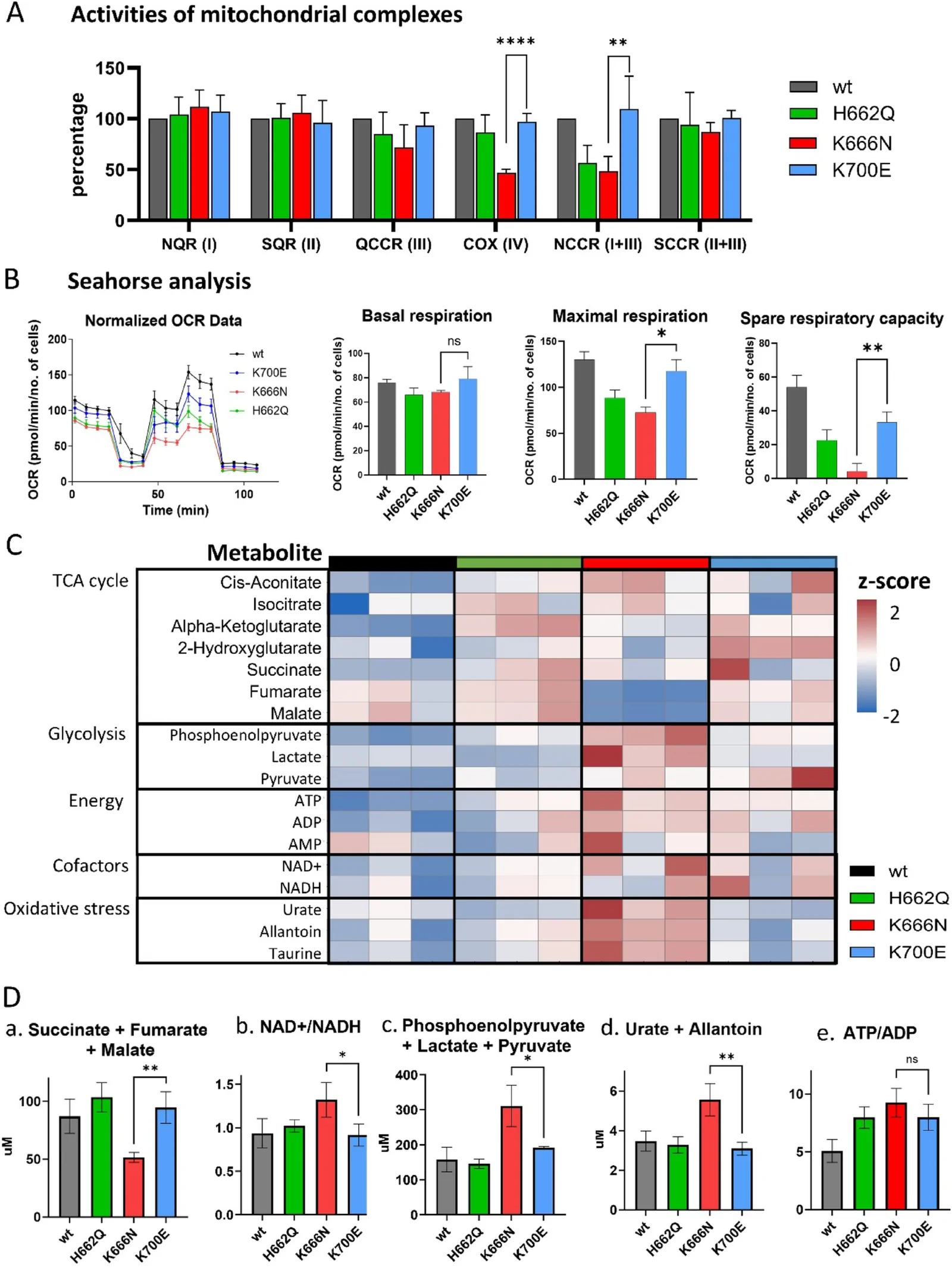
Mitochondrial activity and metabolism in isogenic NALM-6 cell models. (A) Activities of individual mitochondrial respiratory chain complexes—complex I (NADH: ubiquinone oxidoreductase, NQR), complex II (succinate: CoQ reductase, SQR), complex III (ubiquinol: cytochrome c oxidoreductase, QCCR), and complex IV (cytochrome c oxidase, COX) — as well as combined activities of complex I + III (NADH: cytochrome c reductase, NCCR) and complex II + III (succinate: cytochrome c reductase, SCCR). The activities were measured spectrophotometrically and normalized to the wt value (mean ± SD, *n* = 4). (B) Seahorse analysis of the real-time oxygen consumption rate (OCR) and quantification of basal respiration, maximal respiration, and spare respiratory capacity (mean ± SD, *n* = 3). The values are normalized to the number of cells. (C) Heatmap of relative metabolite abundance across clones, expressed as z scores (red indicates metabolite levels above the cross-sample average, and blue indicates levels below it). Metabolites include intermediates of the TCA cycle, glycolysis, pyridine cofactors, energy-related molecules, and oxidative stress markers. Metabolite concentrations were measured by LC/MS in triplicate. (D) Quantification of selected metabolic ratios or sums (mean ± SD, *n* = 3): (a) succinate + fumarate + malate, (b) NAD⁺/NADH, (c) phosphoenolpyruvate + pyruvate + lactate, (d) urate + allantoin, and (e) ATP/ADP. *p < 0.05, **p < 0.01, ****p < 0.0001, ns - nonsignificant

Spectrophotometric activity assays (Fig. 6A) revealed significantly reduced activity of complex IV (cytochrome c oxidase, COX) in K666N cells (~ 50% of other clones), which was consistent with previous Western blot results. Activity of NADH: cytochrome c reductase (NCCR)—reflecting electron transfer through complex I and complex III via coenzyme Q—was also reduced to ~ 50% in both the K666N and H662Q clones.

Seahorse analysis confirmed pronounced mitochondrial dysfunction in K666N cells (Fig. 6B), which significantly reduced maximal respiration (*p* = 0.012) and spare respiratory capacity (*p* = 0.009) while maintaining a basal oxygen consumption rate (OCR) comparable to that of the wild-type. H662Q cells showed a mild decrease in respiratory parameters, whereas K700E cells remained closest to the wild-type profile.

To further investigate these alterations, targeted LC/MS metabolomic profiling was performed. K666N cells presented the most pronounced metabolic shifts across all metabolite categories, including those of TCA intermediates, glycolysis-related metabolites, redox cofactors, energy carriers, and oxidative stress markers (Fig. 6C). The selected metabolite levels are shown in Fig. 6D. Notably, a stepwise depletion of TCA intermediates (from cis-aconitate to malate) was evident in K666N, suggesting impaired TCA flux or downstream metabolite drainage; this was supported by the lowest combined levels of succinate, fumarate, and malate (Fig. 6D-a, *p* = 0.006). Consistent with the phenomenon of impaired electron transport, the NAD⁺/NADH ratio was elevated in K666N cells (Fig. 6D-b, *p* = 0.042), indicating a shift toward a more oxidized intracellular redox state. The total pool of glycolytic intermediates (phosphoenolpyruvate, pyruvate, and lactate) was greatest in K666N cells (Fig. 6D-c, *p* = 0.024), suggesting increased compensatory glycolysis. Concurrently, oxidative stress markers (urate + allantoin) were significantly increased in K666N (Fig. 6D-d, *p* = 0.007). Although the ATP/ADP ratio slightly increased in K666N, this difference was not statistically significant (Fig. 6D-e, *p* > 0.05) and should be interpreted with caution.

Taken together, these findings demonstrate that among the tested *SF3B1* variants, K666N causes the most severe metabolic dysregulation, characterized by combined respiratory chain dysfunction, impaired coenzyme Q-mediated electron transfer, redox imbalance, glycolytic accumulation, and oxidative stress.

## Discussion

*SF3B1* mutations are among the most common splicing factor mutations in MDS and are generally associated with favorable outcomes, particularly in patients with the canonical K700E variant [14]. However, growing evidence—including our current study—suggests that not all *SF3B1* hotspot variants behave equivalently. For example, Dalton et al. reported that the K666N variant is associated with distinct splicing patterns and a less favorable prognosis [13]. Here, we explored the variant-specific biological consequences of distinct *SF3B1* variants in both patient samples and isogenic cell lines, focusing on splicing patterns, transcriptomic alterations, and mitochondrial function.

Our findings extend the established view that A3SS events are a hallmark of *SF3B1*-mutated splicing dysregulation in MDS, chronic lymphocytic leukemia, and uveal melanoma [19, 20]. However, the A3SS represents only one facet of a broader splicing landscape. Additional abnormalities—including RI, SE, and MXE—were also prominent in our dataset, with RI notably elevated in K666 and H662 variants. This aligns with trends observed in spliceosome-mutant hematopoietic cells, where increased RI and SE have been described not only in *SF3B1*-mutated MDS but also in *SRSF2*- and *U2AF1*-mutated contexts [9, 21, 22].

While many aberrant transcripts induced by *SF3B1* mutations contain frameshifts or premature termination codons and may be subject to NMD [9], the engagement of NMD is context-dependent and does not uniformly result in gene-level transcript depletion. Consistent with this notion, variant-specific splicing alterations in our patient CD34⁺ progenitor samples were only rarely accompanied by concordant differential gene expression, indicating that the primary impact of *SF3B1* variants is exerted at the level of isoform usage rather than through widespread NMD-mediated downregulation. This interpretation is further supported by prior studies showing that mis-spliced isoforms can either be degraded or remain stable depending on transcript features and cellular context [22, 23].

Comparative transcriptomic analysis revealed distinct immunoregulatory signatures when directly comparing K700E and K666-variant samples. Relative to K700E, K666-variant samples exhibited comparatively lower expression of immunoglobulin transcripts alongside higher expression of HLA genes, indicating a variant-specific shift in immune transcriptional identity. These differences were accompanied by enhanced interferon-related and innate inflammatory signaling programs in the K666-variant context. These findings expand on previous reports showing that *SF3B1* mutations broadly activate immune and inflammatory pathways in MDS [24, 25]. One mechanism involves the accumulation of mis-spliced RNAs that activate cytosolic sensors such as RIG-I, triggering interferon and NF-κB signaling [26], which may drive HLA expression [27]. Additionally, *MAP3K7*—a key NF-κB regulator mis-spliced in an *SF3B1* variant-specific manner [28, 29]—displayed isoform differences between K700E and K666-variant in our dataset. The immune profile observed in K666-variant progenitors is therefore best interpreted as reflecting altered lineage priming, characterized by relative attenuation of B-cell–associated transcriptional programs and increased expression of myeloid or antigen-presenting markers. Whether this pattern represents compensatory immune activation or a signature of impaired immune surveillance in the context of a more aggressive *SF3B1* variant remains unclear. Nonetheless, our data indicate that individual *SF3B1* variants distinctly reprogram immune signaling, thus reinforcing the need for variant-informed immunogenomic stratification in MDS.

Our patient RNA-seq analyses were designed to capture broad variant-level trends rather than definitive subtype-specific effects. Given the imbalance between K700E and the pooled K666-variant group, as well as the compositional heterogeneity of the latter—including recurrent co-mutations in genes with known roles in transcriptional and splicing regulation—statistical power to resolve K666-variant–specific signals was necessarily limited, as is typical for real-world clinical genomics datasets. To address these constraints, we complemented patient-based analyses with genetically defined isogenic cell line models, which allowed controlled dissection of mutation-specific effects in the absence of clinical and genetic confounders.

The most striking finding emerging from our patient transcriptomic analyses was a variant-specific alteration of mitochondrial gene expression, which was subsequently validated and mechanistically explored using isogenic cell line models. This finding aligns with growing evidence linking spliceosomal mutations to mitochondrial dysfunction. *SF3B1* mutations have been shown to alter mitochondrial gene expression, impair respiratory chain complex assembly, and reprogram metabolism [18]. Detailed metabolic characterization revealed that mutant *SF3B1* decreases mitochondrial respiration and promotes glycolysis to compensate for defective mitochondrial metabolism [30]. Similar dysfunctions have been reported for *SRSF2* and *U2AF1*, which affect mitophagy, redox balance, and mtDNA maintenance [31, 32].

Our data confirmed severe dysfunction of mitochondrial bioenergetics in *SF3B1*-mutated cells, most prominently in the K666N isogenic model. Reduced levels of several OXPHOS subunits were not consistently associated with direct splicing alterations of the corresponding transcripts, supporting an indirect mechanism downstream of variant-specific splicing programs rather than direct missplicing of individual OXPHOS genes. These changes were not attributable to differences in mtDNA content, indicating a regulatory rather than biogenetic basis. In K666N cells, we observed complex IV deficiency, which was supported by multiple findings, including reduced expression of COX subunits, impaired holocomplex IV assembly, and decreased enzymatic activity. Nevertheless, Seahorse analysis revealed comparable basal respiration clones in all cell lines, suggesting preserved OXPHOS under nutrient-rich conditions. However, all the mutant clones presented reduced maximal respiration rate and spare respiratory capacity. In particular, K666N cells exhibited minimal respiratory reserve, operating near its respiratory limit even under basal conditions. This was accompanied by decreased fumarate and malate, suggesting impaired TCA flux [33]. Metabolomic profiling showed glycolytic metabolite accumulation in K666N, indicating glycolytic shift as a compensation for limited TCA. Substantially increased lactate level suggests compensatory NADH reoxidation via pyruvate-to-lactate conversion [34]. Thus, elevated NAD⁺/NADH and ATP levels likely reflect glycolytic compensation. Furthermore, increased oxidative stress markers in the K666N clone suggest metabolic stress, potentially arising from complex IV dysfunction and the production of reactive oxygen species (ROS) [35, 36].

The H662Q clone presented an intermediate metabolic phenotype, with partial OXPHOS impairment but preserved energy balance. The Seahorse assay revealed moderate respiratory defects, and NCCR activity was reduced despite normal individual complex I and III activity. This finding suggests impaired coenzyme Q–mediated electron transfer, which is a defect known to compromise efficiency and increase ROS [37, 38]. However, despite partial OXPHOS dysfunction, H662Q did not shift toward glycolysis, possibly due to compensatory stress‒response pathways. These findings underscore that not all *SF3B1* variants induce metabolic reprogramming to the same extent.

The mitochondrial defects observed in K666N cells may be mechanistically linked to aberrant splicing of genes critical for mitochondrial function. For example, *COA8* is involved in complex IV stability and its deficiency has been associated with isolated complex IV deficiency [39]; *FOXRED1* is required for proper complex I assembly [40]; *TAFAZZIN* participates in cardiolipin remodeling [41], which is essential for mitochondrial membrane organization and respiratory supercomplex stability; and *COASY* and *NAPRT* contribute to coenzyme A and NAD⁺ metabolism, both of which are vital for mitochondrial energy production [42, 43]. Notably, recent evidence links COASY mis-splicing caused by *SF3B1* mutations to impaired heme biosynthesis and erythroid differentiation in MDS-RS [44]. Taken together, the mis-splicing of these genes may compromise OXPHOS integrity. Additionally, dysfunction of complexes I and IV may compromise the mitochondrial membrane potential and retrograde signaling [45], leading to mitochondrial stress and the secondary downregulation of mitochondrial transcription evident in our data.

It should be noted, however, that while these associations provide a plausible mechanistic framework, direct causal links between individual splicing events and specific mitochondrial phenotypes remain to be experimentally validated. In particular, the lack of in vivo validation represents a limitation of the present study. Nonetheless, the use of genetically defined isogenic NALM-6 cell line models enabled controlled interrogation of *SF3B1* variant-specific effects in a uniform genetic background, independent of clinical and mutational heterogeneity. Although NALM-6 cells are of B-lymphoblastoid origin and do not model erythroid differentiation, they represent a spliceosome-competent and experimentally tractable system. Moreover, the consistency of splicing and metabolic phenotypes across patient-derived and engineered samples supports the biological relevance of our observations. In addition, our transcriptomic data demonstrate that several canonical *SF3B1*-associated genes linked to mitochondrial iron metabolism, including *ABCB7*, *PPOX*, and *TMEM14C*, are detectably expressed in NALM-6 cells. This finding indicates that *SF3B1*-driven perturbations affecting mitochondrial and iron-handling pathways are not strictly erythroid-restricted, even though the disease-defining ring sideroblast phenotype likely arises from erythroid-specific downstream regulatory programs.

Another limitation of our study, as with many isogenic model–based investigations, is that functional mitochondrial assays were performed using a single independently edited clone per genotype. In addition, the absence of isoform-specific rescue experiments—such as re-introduction of wild-type *SF3B1* into the K666N background—precludes definitive attribution of all observed mitochondrial phenotypes solely to the *SF3B1* variant. These constraints should be taken into account when interpreting downstream phenotypic effects. Accordingly, future studies employing additional cell lineages or in vivo model systems will be important to further validate and generalize the present findings.

Clinically, our findings raise important questions about the current classification and risk stratification of *SF3B1*-mutated MDS. While K700E is considered a prototypically “favorable” variant [14], the more pronounced molecular and mitochondrial defects in non-K700E cells suggest distinct behaviors and prognostic impacts. Our results support a refined view of *SF3B1* mutations not as a uniform molecular entity, but rather as a heterogeneous spectrum with divergent consequences. These findings also underscore the therapeutic potential of targeting metabolic vulnerabilities, especially in patients harboring aggressive K666N variant. Given that different *SF3B1* variants appear to exhibit variant-specific molecular profiles, incorporating the *SF3B1* mutation variants into clinical decision-making may be warranted. Notably, Adema et al. [46] reported that the presence of the H662 variant may reduce the lenalidomide response. Our data suggest that additional therapeutic strategies—such as direct OXPHOS targeting, pharmacological induction of mitochondrial stress, glycolysis inhibition, or modulation of NAD⁺ metabolism—could be explored as precision medicine approaches in selected *SF3B1*-mutated subgroups. Finally, the distinct mitochondrial bioenergetics observed among *SF3B1* variants emphasize the potential utility of metabolic profiling and functional assays as complementary tools for subclassifying *SF3B1*-mutated MDS and tailoring treatment accordingly.

## Conclusions

In conclusion, our integrated analysis revealed considerable heterogeneity among *SF3B1* hotspot variants. The distinct molecular background and mitochondrial bioenergetics associated with the K700E, K666N, and H662Q hotspots underscore the need for variant-specific evaluation in both research and clinical settings. These findings provide a rationale for tailored therapeutic approaches and support more nuanced *SF3B1* mutation stratification in MDS.

## Supplementary Information


Supplementary Material 1.


## Data Availability

Raw NGS data from RNA sequencing were deposited in the NCBI SRA database (BioProject ID PRJNA1291605).
