# Supplementary material for: Variant-specific SF3B1 mutations drive distinct splicing and mitochondrial dysfunction in myelodysplastic neoplasms
Source: Hum Genomics. 2026 Jun 18;20:130. doi: 10.1186/s40246-026-01000-2 (PMC13523384; doi:10.1186/s40246-026-01000-2)
Supplement: Supplementary file 1 [file 40246_2026_1000_MOESM1_ESM.pdf]

Variant-specific *SF3B1* mutations drive distinct splicing and mitochondrial dysfunction in myelodysplastic neoplasms

Andrea Hruštincová, Iva Trsová, David Kundrát, Monika Beličková, Monika Kaisrlíková, Jitka Veselá, Zdeněk Krejčík, Alžběta Hlaváčková, Jiří Suttnar, Hana Štufková, Nikol Volfová, Hana Hansíková, Tatiana Aghová, Zuzana Zemanová, Anna Jonášová, Jaroslav Čermák, Markéta Šťastná Marková, and Michaela Dostálová Merkerová

SUPPLEMENTARY INFORMATION

**Supplementary Materials and Methods**	Page 2
**Supplementary Tables S1–S5**	Page 5
**Supplementary Figures S1–S5**	Page 10
**References**	Page 15

SUPPLEMENTARY MATERIALS AND METHODS

Patients. A total of 121 MDS patients with *SF3B1* mutations who were diagnosed after 2012 and continuously followed up at the Institute of Hematology and Blood Transfusion and the General University Hospital in Prague were included. Another 32 mutation-negative patients were enrolled for comparison. Diagnoses were classified according to the WHO 2022 criteria [1], and prognostic risk was assessed via the International Prognostic Scoring System (IPSS-M) [2]. None of the patients had a prior malignancy. Bone marrow samples from 13 healthy donors (6 males, 7 females; mean age 41 years (range 27–69)) served as controls for RNA sequencing. All participants provided written informed consent. The study was approved by the Institutional Scientific Board and Ethics Committee in accordance with the Declaration of Helsinki.

Cell lines. Four NALM-6 cell lines were used: one with wild-type *SF3B1* and three CRISPR/Cas9-edited mutants (K700E, K666N, H662Q; Horizon Discovery, Cambridge, UK). According to the supplier, these variants were introduced by endogenous genome editing at the *SF3B1* locus, and the resulting cell lines were genetically validated prior to delivery. We verified the presence of the engineered *SF3B1* variants in the NALM-6 clones by Sanger sequencing and confirmed karyotypes by standard cytogenetic analysis. Cells were cultured in RPMI-1640 medium supplemented with 10% fetal bovine serum (Thermo Fisher Scientific) at 37 °C in 5% CO_2. All cell lines were confirmed to be mycoplasma-free prior to use (MycoAlert PLUS, Lonza, Basel, Switzerland).

Cell separation and nucleic acid extraction. Mononuclear cells (MNCs) were isolated from BM aspirates via Ficoll–Paque density gradient centrifugation (GE Healthcare, Munich, Germany). CD34+ cells were further enriched by magnetic separation (Miltenyi Biotec, Bergisch Gladbach, Germany). Total RNA was extracted using the acid guanidinium–phenol–chloroform method, and residual genomic DNA was removed by DNase I treatment (Qiagen, Hilden, Germany). The RNA concentration and integrity were assessed using a Qubit 3 fluorometer (Thermo Fisher Scientific, Waltham, MA, USA) and an Agilent 4200 TapeStation (Agilent Technologies, Santa Clara, CA, USA), respectively.

Mutational screening. Targeted next-generation sequencing (NGS) was performed using either the TruSight Myeloid Sequencing Panel (Illumina, San Diego, CA, USA; 54 genes, 568 amplicons) or a custom SureSelect XT HS2 panel (Agilent; 59 genes, 442 amplicons). Libraries were sequenced on an Illumina MiSeq platform. Variants with coverage ≥ 300 and variant allele frequency (VAF) ≥ 2% were retained. Variant annotation and interpretation were performed using multiple databases (1000 Genomes, dbSNP, Varsome, ExAC) via an in-house bioinformatics pipeline.

RNA sequencing and bioinformatic analysis. Ribosomal RNA was depleted using the RiboCop rRNA Depletion Kit (Lexogen, Vienna, Austria). cDNA libraries were prepared with the NEBNext Ultra II RNA Library Prep Kit (New England Biolabs, Ipswich, MA, USA). Paired-end sequencing (2×100 bp) was performed on an Illumina NovaSeq X. Sequence reads were quality-checked with FastQC [3] and trimmed with Trimmomatic [4]; reads were aligned to the human genome (hg38) using STAR [5]. Gene expression was quantified with StringTie [6]. Principal component analysis (PCA) was performed using the pcaMethods R package to assess sample clustering. Alternative splicing was analyzed with rMATS [7], which detects five major types of splicing events: skipped exons (SE), retained introns (RI), alternative 5′ and 3′ splice sites (A5SS, A3SS), and mutually exclusive exons (MXE). Alternative splicing was quantified as percent spliced in (PSI), and group differences were expressed as the ΔPSI. Events with a ΔPSI and FDR < 0.1 were considered significant, a threshold commonly applied in splicing-focused analyses to balance sensitivity and specificity in moderate sample sizes. Differential gene expression was assessed with edgeR, which requires transcripts to have ≥ 5 counts per million (CPM) in ≥ 50% of samples and a false discovery rate (FDR) < 0.05. Functional enrichment was analyzed using

DAVID [8] and gene set enrichment analysis (GSEA) [9]. Data visualization was performed using ggplot2 and pheatmap.

Mitochondrial DNA quantification. Total DNA was extracted from cells using the QIAamp DNA Mini Kit (Qiagen). The mitochondrial DNA (mtDNA) content was quantified by real-time PCR (CFX96 Real-Time System; Bio-Rad, Hercules, CA, USA) using primers for the mitochondrial gene *MT-RNR2* and the nuclear gene *GAPDH* as previously described [10, 11]. The relative mtDNA content was calculated as the *MT-RNR2*/*GAPDH* ratio.

Electrophoresis and Western blot. SDS–PAGE and Blue Native PAGE (BN–PAGE) were performed as previously described [10]. For SDS–PAGE, cell lysates were prepared in RIPA buffer, and 5–15 µg of protein was separated on 12% polyacrylamide gels (Bio-Rad). For BN–PAGE, mitochondria were isolated via differential centrifugation [12]. The mitochondrial fraction was solubilized with n-dodecyl β-d-maltoside (DDM) at a final protein ratio of 4.5 mg DDM/mg in a buffer containing 1.5 mM aminocaproic acid, 0.05 M Bis-Tris, and 0.5 M EDTA, and 15 µg of mitochondrial protein was loaded onto 6–15% gradient gels. Proteins were transferred to PVDF membranes via semi-dry transfer (Bio-Rad), blocked in 2% nonfat milk and probed with primary and HRP-conjugated secondary antibodies. The blots were developed with chemiluminescent substrate (SuperSignal West Femto or Pico PLUS, Thermo Fisher Scientific) and imaged on a G:BOX system (Syngene, Bangalore, India). Actin and tubulin were used as loading controls; VDAC1 was used as a mitochondrial marker. In BN-PAGE, complex II served as an internal reference. The antibody details are listed in ***Table 1.***

Table 1. Antibodies employed in this study.

Antibody	Complex	Dilution	Company	Product number
TUBULIN	-	1:2 000	Cell Signaling (Danvers, MA, USA)	2125S
ACTIN	-	1:2 000	Cell Signaling	4970S
VDAC1	-	1:2 000	Abcam (Cambridge, UK)	Ab14734
NDUFB6	Complex I	1:3 333	Abcam	Ab110244
NDUFS2	Complex I	1:2 000	Abcam	Ab110249
NDUFA9	Complex I	1:3 333	Abcam	Ab14713
NDUFB8	Complex I	1:5 000	Abcam	Ab110242
SDH70	Complex II	1:20 000	Abcam	Ab14715
SDH30	Complex II	1:5 000	Abcam	Ab14714
CORE1	Complex III	1:2 000	Abcam	Ab110252
CORE2	Complex III	1:3 333	Abcam	Ab14745
COX1	Complex IV	1:4 000	Abcam	Ab14705
COX2	Complex IV	1:10 000	Abcam	Ab110258
COX5a	Complex IV	1: 10 000	Abcam	Ab110262
ATP5A	Complex V	1:5 000	Abcam	Ab14748
ATP5B	Complex V	1:10 000	Abcam	Ab14730

Respiratory chain enzyme activities. The activities of respiratory chain complexes (RCCs) — including complex I (NADH:ubiquinone oxidoreductase, NQR), complex II (succinate:CoQ reductase, SQR), complex III (ubiquinol:cytochrome c oxidoreductase, QCCR), and complex IV (cytochrome c oxidase, COX), as well as the combined activities of complex I + III (NADH:cytochrome c reductase, NCCR) and complex II + III (succinate:cytochrome c reductase, SCCR), were measured spectrophotometrically at 37 °C using a UV/VIS system (Shimadzu, Japan) as previously described by Rodinova et al. [13] All RCC activities were normalized to citrate synthase (CS) activity.

Seahorse metabolic analysis. Mitochondrial respiration was measured via a Seahorse XFp Analyzer (Agilent) with a Cell Mito Stress Test Kit. The cells (150,000/well) were seeded in PDL-coated miniplates with XF RPMI Base Medium (pH 7.4) containing 10 mM glucose, 1 mM pyruvate, and 2 mM glutamine.

The final concentrations of the injected compounds were 1 µM oligomycin, 0.5 µM FCCP, and 0.5 µM rotenone + antimycin A. All reagents were purchased from Agilent.

Targeted metabolite profiling. Metabolites from the tricarboxylic acid (TCA) cycle (cis-aconitate, isocitrate, α-ketoglutarate, 2-hydroxyglutarate, succinate, fumarate, malate), glycolysis (lactate, pyruvate, phosphoenolpyruvate), NAD^+, NADH, ATP, ADP, AMP, and oxidative stress markers (urate, allantoin, taurine) were quantified by liquid chromatography/mass spectrometry (LC/MS). Metabolites were extracted from 1 × 10^6 cells in 1000 µL of methanol:acetonitrile:water (5:3:2, v/v/v), dried under vacuum, and reconstituted in 100 µL of water. The samples were separated on a Prominence HPLC system (Shimadzu, Kyoto, Japan) and analyzed using a QTRAP 4000 mass spectrometer (SCIEX, Framingham, MA, USA). The data were processed with Analyst v1.6 as previously described [14].

Statistical analysis. Statistical analyses were performed in GraphPad Prism 10 (GraphPad Software, La Jolla, CA, USA). Group comparisons were made using Student's t-test and ANOVA. Progression-free survival (PFS) was analyzed by Kaplan–Meier plots with the log-rank test. Because the overall survival results were similar, only PFS data are shown. Multivariable survival analysis was not performed because of the limited size of the patient subgroups, which would preclude reliable estimation. The threshold for statistical significance was $p < 0.05$.

SUPPLEMENTARY TABLES

Supplementary Table 1. Characteristics of the cohort.

	***SF3B1*-mutated patients**	**Wild-type patients**
No. of patients	121	32
Sex (male/female)	67/54	13/19
Age, years (mean [range])	70 (46–89)	60 (27–78)
Diagnosis (SF3B1/5q/biTP53/LB/IB1/IB2) *	74/11/3/6/14/13	0/10/0/17/2/3
IPSS-M category (very low/low/mod. low/mod. high/high/very high)	18/39/24/12/15/13	12/14/1/3/2/0
$SF3B1^{5q}$ / $SF3B1^{\beta}$ / $SF3B1^{\alpha}$ #	10/28/83	n.a.
Marrow blasts [%] (mean [range])	4.0 (0.0–19.0)	5.1 (0.0–19.8)
Hemoglobin [g/L] (mean [range])	93 (38–135)	109.9 (38–141)
Neutrophils [$\times10^9$/L] (mean [range])	3.0 (0.2–8.1)	2.9 (0.2–8.1)
Platelets [$\times10^9$/L] (mean [range])	268 (27–970)	209 (27–970)

n.a. – not applicable

*Diagnoses were classified based on the 2022 WHO criteria [1]. SF3B1 - MDS with low blasts and *SF3B1* mutation, 5q - MDS with low blasts and isolated 5q deletion, biTP53 - MDS with biallelic *TP53* inactivation, LB - MDS with low blasts, IB1 - MDS with increased blasts 1, IB2 - MDS with increased blasts 2.

#Categories based on the publication of Bernard et al. [2]: $SF3B1^{5q}$ - mutant *SF3B1* with isolated del(5q), $SF3B1^{\beta}$ - mutant *SF3B1* cooccurring with *BCOR, BCORL1, NRAS, RUNX1, SRSF2,* or *STAG2* mutations, and $SF3B1^{\alpha}$ - mutant *SF3B1* with any other mutation or as an isolated event.

Supplementary Table 2. Distribution of recurrent co-mutations and cytogenetic abnormalities the full clinical *SF3B1*-mutated cohort.

	**All *SF3B1*-mut MDS (n = 121)**	**MDS-*SF3B1* (n = 74)**	**Other *SF3B1*-mut MDS (n = 47)**	**K700E (n = 65)**	**Pooled K666-variant (n = 20)**
**Co-mutations**					
TET2	35	26	9	21	4
DNMT3A	26	18	8	17	2
RUNX1	18	7	11	15	3
ASXL1	14	10	4	5	4
SRSF2	5	3	2	1	2
U2AF1	2	1	1	1	0
ZRSR2	4	1	3	1	3
**Major cytogenetic abnormalities**					
Normal karyotype	68	56	12	36	9
del(5q)	23	0	23	16	1
+8	10	6	4	9	0
Complex karyotype	8	0	8	2	3
−7/del(7q)	2	0	2	2	0

Pooled K666-variant group includes K666N, K666E, K666R, K666T, and K666M across the full clinical *SF3B1*-mutated cohort.

Supplementary Table 3. Characteristics of K700E and K666-variant *SF3B1*-mutated patients included in the RNA-seq analysis

	**K700E**	**K666-variant**
No. of patients	30	10
Sex (male/female)	19/11	8/2
Age, years (mean [range])	71 (59–89)	70 (60–78)
Diagnosis (SF3B1/5q/biTP53/LB/IB1/IB2) *	18/4/0/2/4/2	5/0/0/1/2/1
IPSS-M category (very low/low/mod. low/mod. high/high/very high)	5/13/5/1/5/1	1/3/4/1/1/1
$SF3B1^{5q}$ / $SF3B1^{\beta}$ / $SF3B1^{\alpha}$ #	4/4/22	0/2/8
Marrow blasts [%] (mean [range])	4.5 (0.0–15.0)	7.6 (0.2–19.0)
Hemoglobin [g/L] (mean [range])	94 (71–135)	97 (67–120)
Neutrophils [$\times 10^9$/L] (mean [range])	2.9 (0.3–8.1)	2.6 (0.5–7.7)
Platelets [$\times 10^9$/L] (mean [range])	260 (27–970)	219 (94–415)
SF3B1 VAF [%] (mean [range])	42 (26–48)	32 (9–42)
≥1 additional mutation	23	6
Cytogenetics (normal / abnormal)	14/16	7/3
Complex karyotype	1	1

*Diagnoses were classified based on the 2022 WHO criteria [1]. SF3B1 - MDS with low blasts and *SF3B1* mutation, 5q - MDS with low blasts and isolated 5q deletion, biTP53 - MDS with biallelic *TP53* inactivation, LB - MDS with low blasts, IB1 - MDS with increased blasts 1, IB2 - MDS with increased blasts 2.

#Categories based on the publication of Bernard et al. [2]: $SF3B1^{5q}$ - mutant *SF3B1* with isolated del(5q), $SF3B1^{\beta}$ - mutant *SF3B1* cooccurring with *BCOR, BCORL1, NRAS, RUNX1, SRSF2,* or *STAG2* mutations, and $SF3B1^{\alpha}$ - mutant *SF3B1* with any other mutation or as an isolated event.

Supplementary Table 4. Functional enrichment of alternatively spliced genes between patient samples with K700E or K666-variants. A total of 711 genes showing significant differences in isoform inclusion between the two *SF3B1* variant groups (ΔPSI > 0.1, FDR < 0.1) were subjected to functional annotation using the DAVID tool. The table lists the five most significantly enriched Gene Ontology Biological Process (GO BP) terms, along with genes contributing to each term and their corresponding enrichment p values.

GO PB Process	GO ID	Genes	p value
**DNA repair**	GO:0006281	*BRCA1, BRIP1, DCLRE1C, POLG, POLL, TOP3A, EP400, FAN1, INO80E, KIN, MRNIP, NSMCE4A, RAD50, RAD51D, REV1, RECQL4, RUVBL2, XRCC3, APTX, APEX1, CEP164, CHD1L, ENDOV, MACROH2A1, MORF4L2, NONO, NFRKB, NPM1, RRM1*	4,0E-8
**DNA recombination**	GO:0006310	*BRCA1, DCLRE1C, TOP3A, INO80E, KIN, RAD50, RAD52, RECQL4, RUVBL2, XRCC3, APEX1, NONO, NFRKB*	2,4E-6
**Telomere maintenance**	GO:0000723	*ACD, ATM, CTC1, DCLRE1C, INO80E, RAD50, RAD51D, RECQL4, RUVBL2, APEX1, NFRKB*	9,1E-6
**Double-strand break repair via homologous recombination**	GO:0000724	*ATM, BRCA1, POLL, TOP3A, FAN1, NSMCE4A, RAD50, RAD51D, RAD52, RECQL4, XRCC3, AP5Z1, ZSWIM7*	6,1E-5
**Regulation of cell cycle**	GO:0051726	*ATM, BRCA1, EP400, HBP1, INO80E, L3MBTL1, MEAF6, RAD51D, RASSF1, RUVBL2, TSC2, BTRC, CCNL2, CDK10, MORF4L2, NFRKB, SIRT2, TFDP1, TFDP2, TBRG1*	9,1E-5

Supplementary Table 5. Number of genes whose expression was significantly altered in a series of differential expression analyses performed between each pair of isogeneic NALM-6 cell lines (wild-type - WT, K700E, K666N, or H662Q). The differential expression analysis settings were as follows: FDR < 0.05 and |logFC| > 1. The color shading in the table highlights differences in the number of dysregulated genes: red indicates a high number, whereas blue indicates a low number.

**No. of genes**	**Compared to:**		
**Upregulated in:**	K666N	H662Q	WT
K700E	264	165	47
K666N		94	148
H662Q			140

**No. of genes**	**Compared to:**		
**Downregulated in:**	K666N	H662Q	WT
K700E	212	143	119
K666N		88	272
H662Q			186

SUPPLEMENTARY FIGURES

Supplementary Figure 1. Kaplan-Meier analysis of progression free survival (PFS) in the MDS patient cohort. (A) PFS in the entire cohort of 153 MDS patients, stratified according to the IPSS-M risk categories [1]. (B) PFS in the subset of *SF3B1*-mutated patients (n = 121), stratified by IPSS-M. (C) PFS in *SF3B1*-mutated patients classified into molecular subgroups according to Bernard et al. [2]: $SF3B1^{5q}$ (*SF3B1* mutation with isolated del(5q)), $SF3B1^{\beta}$ (*SF3B1* mutation cooccurring with *BCOR, BCORL1, NRAS, RUNX1, SRSF2,* or *STAG2*), and $SF3B1^{\alpha}$ (*SF3B1* mutation with any other co-mutation or as an isolated event). For comparison, wild-type (wt) patients (n = 32) were included in the plot.

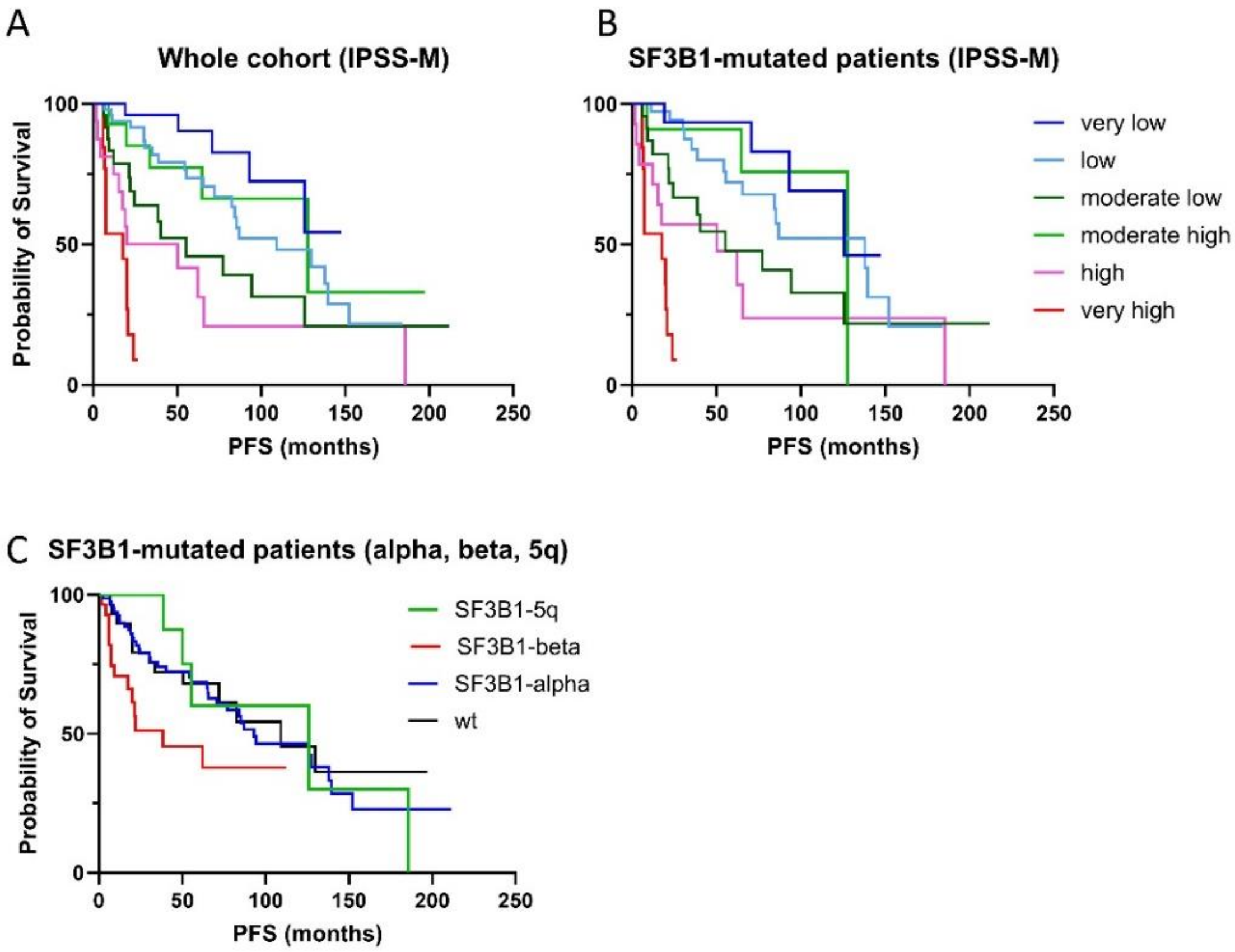

Supplementary Figure 2. Gene expression analysis of K700E and K666-variant patient samples. Heatmap showing the top 50 genes contributing to enrichment of the mitochondrial gene expression pathway in the GSEA output. Expression values are shown as colors (red, higher expression; blue, lower expression) and reflect relative differences between K700E and K666-variant samples, rather than absolute changes relative to mutation-negative or healthy controls.

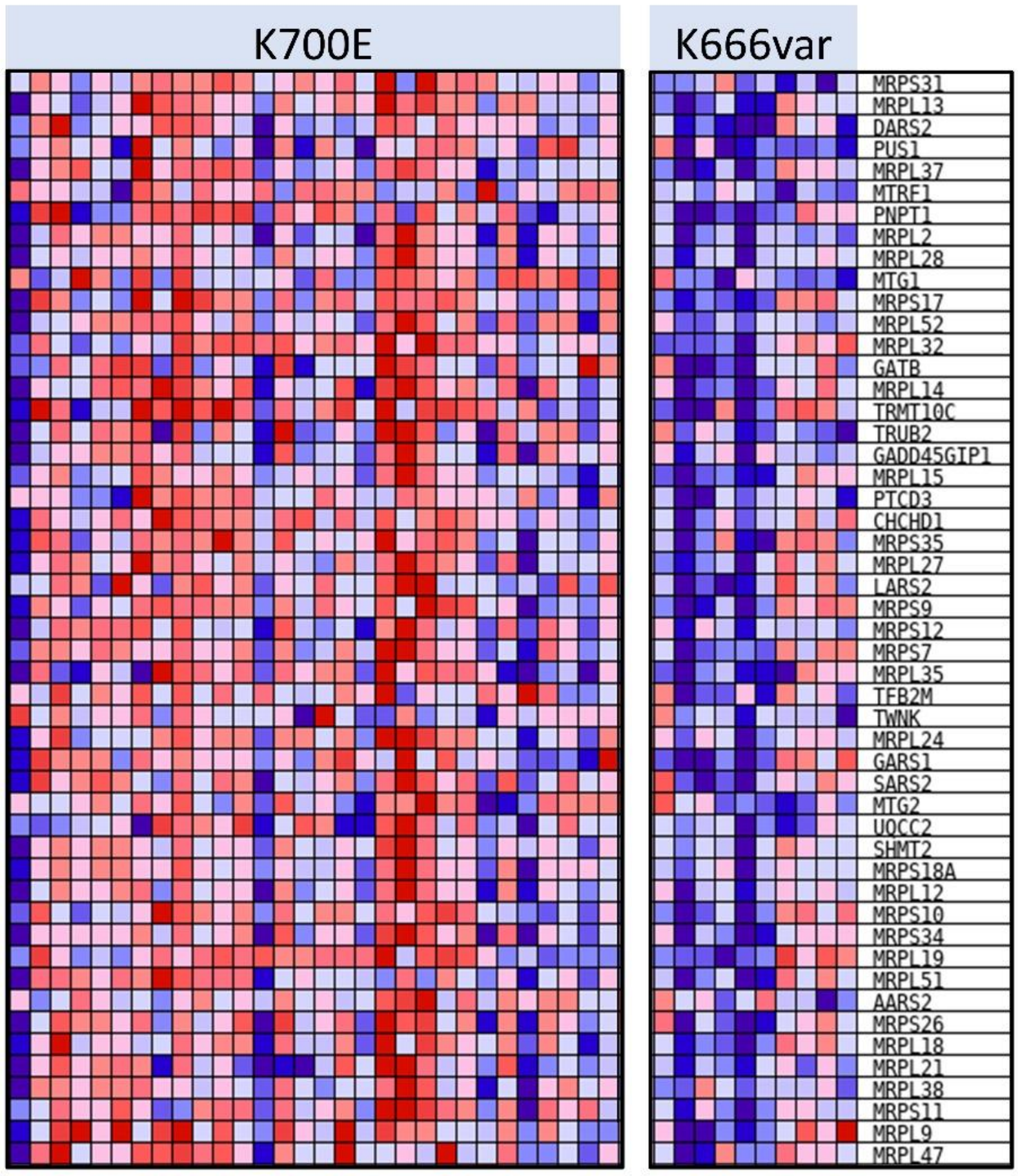

Supplementary Figure 3. Venn diagram illustrating little overlap between genes affected by differential splicing (ΔPSI > 0.1, FDR < 0.1) and those with altered transcript levels in differential expression analysis (FDR < 0.05) in NALM-6 lines with K700E and K666N variants. Up – upregulated expression, down – downregulated expression in K700E compared to K666N cells.

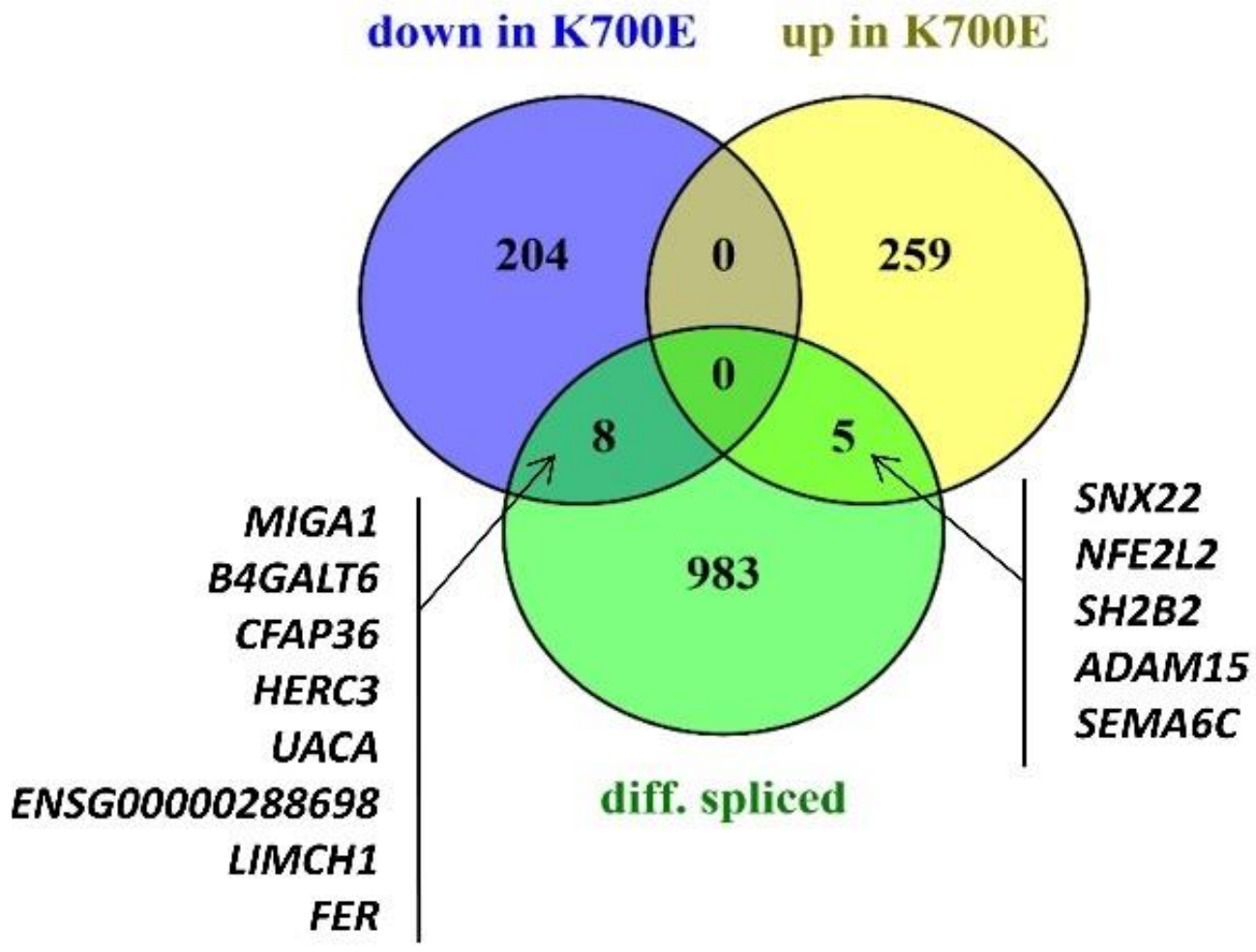

Supplementary Figure 4. Supporting SDS-PAGE Western blot data for OXPHOS subunit analyses shown in Fig. 5B–C. (A) Cropped composite panels derived from three independent biological replicates. (B) Individual full-length, uncropped SDS-PAGE Western blots from a representative experiment. Blots display analyses of OXPHOS complex subunits from complexes I (NDUFB6, NDUFS2, NDUFA9, and NDUFB8), II (SDH30 and SDH70), III (CORE1 and CORE2), IV (COX1, COX2, and COX5A), and V (ATP5A and ATP5B) across isogenic NALM-6 clones (wt, H662Q, K666N, and K700E). Actin and tubulin are shown as loading controls; VDAC1 serves as a mitochondrial outer membrane marker (OMM). A fibroblast sample was included as an additional control (CTR). Individual proteins were identified based on their characteristic migration patterns and were consistently annotated across experiments to ensure correct alignment and comparability between membranes.

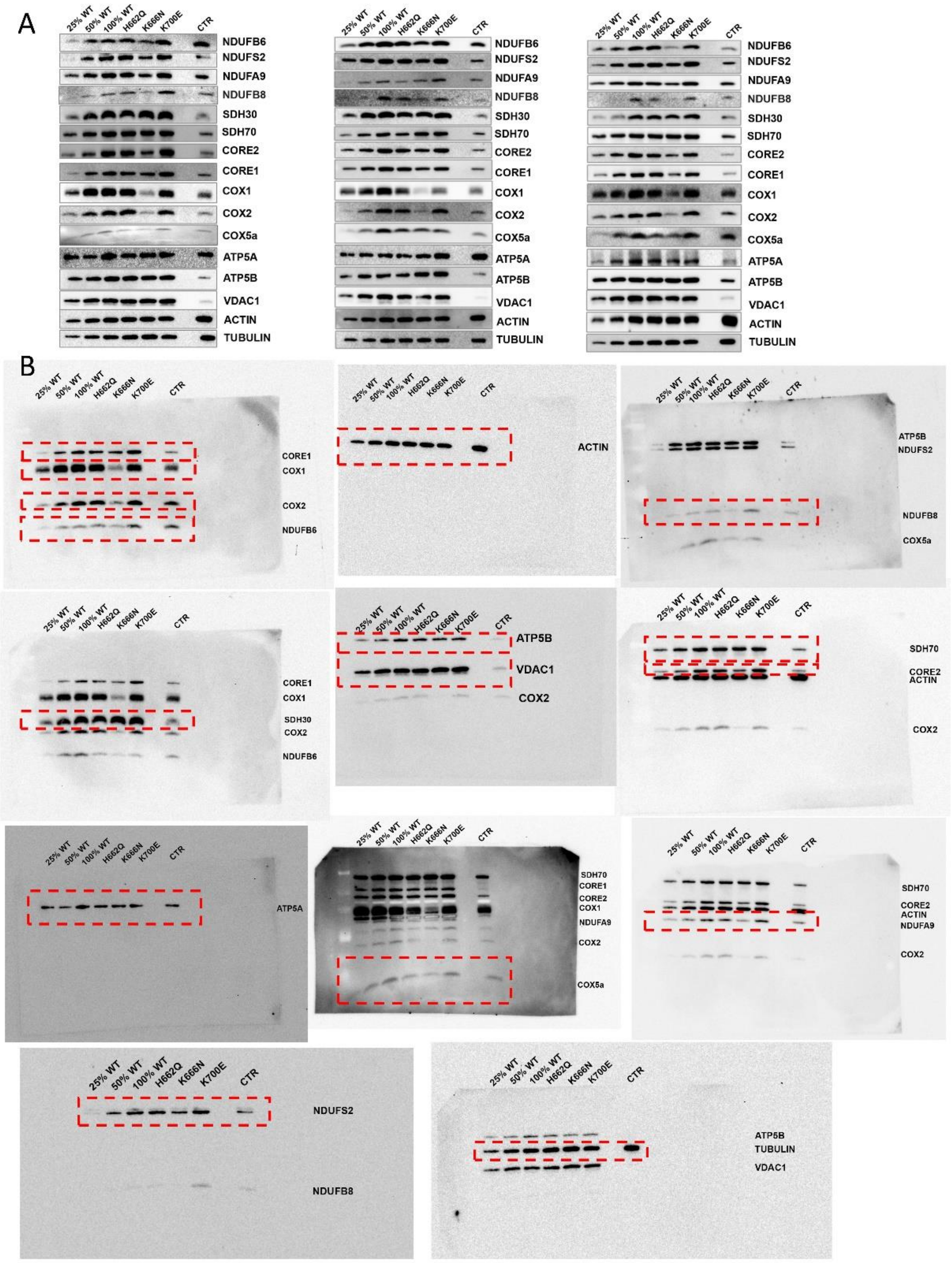

Supplementary Figure 5. Supporting Blue Native PAGE (BN-PAGE) data for OXPHOS complex analyses shown in Fig. 5D–E. (A) Cropped composite panels derived from three independent biological replicates. (B) Individual full-length, uncropped BN-PAGE membranes from one representative experiment. Intact respiratory chain complexes I–V were resolved from isogenic NALM-6 clones (wt, H662Q, K666N, and K700E) and detected by immunoblotting using OXPHOS complex-specific antibodies. Individual complexes were identified based on their characteristic migration patterns and were consistently annotated across experiments to ensure correct alignment and comparability between membranes. Asterisks indicate ATP synthase (complex V) subcomplexes.

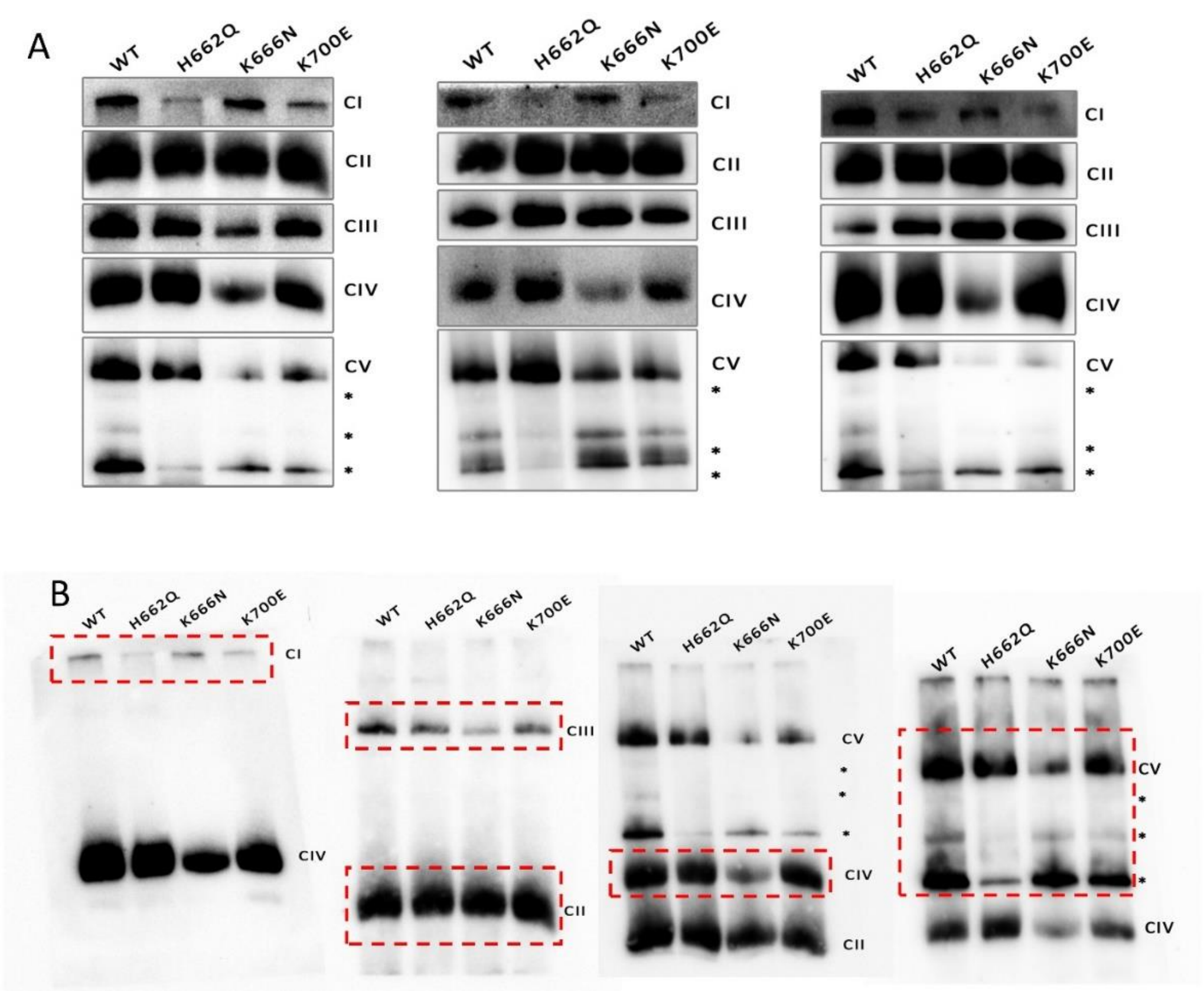

REFERENCES

1. Khoury JD, Solary E, Abla O, et al (2022) The 5th edition of the World Health Organization Classification of Haematolymphoid Tumours: Myeloid and Histiocytic/Dendritic Neoplasms. Leukemia 36:1703–1719
2. Bernard E, Tuechler H, Greenberg PL, et al (2022) Molecular International Prognostic Scoring System for Myelodysplastic Syndromes. NEJM Evid. https://doi.org/10.1056/EVIDoa2200008
3. Andrews S (2010) FastQC - A quality control tool for high throughput sequence data. http://www.bioinformatics.babraham.ac.uk/projects/fastqc/. Babraham Bioinforma. https://doi.org/citeulike-article-id:11583827
4. Bolger AM, Lohse M, Usadel B (2014) Trimmomatic: A flexible trimmer for Illumina sequence data. Bioinformatics. https://doi.org/10.1093/bioinformatics/btu170
5. Dobin A, Davis CA, Schlesinger F, Drenkow J, Zaleski C, Jha S, Batut P, Chaisson M, Gingeras TR (2013) STAR: Ultrafast universal RNA-seq aligner. Bioinformatics. https://doi.org/10.1093/bioinformatics/bts635
6. Pertea M, Pertea GM, Antonescu CM, Chang TC, Mendell JT, Salzberg SL (2015) StringTie enables improved reconstruction of a transcriptome from RNA-seq reads. Nat Biotechnol. https://doi.org/10.1038/nbt.3122
7. Shen S, Park JW, Lu ZX, Lin L, Henry MD, Wu YN, Zhou Q, Xing Y (2014) rMATS: Robust and flexible detection of differential alternative splicing from replicate RNA-Seq data. Proc Natl Acad Sci U S A 111:E5593–E5601
8. Sherman BT, Hao M, Qiu J, Jiao X, Baseler MW, Lane HC, Imamichi T, Chang W (2022) DAVID: a web server for functional enrichment analysis and functional annotation of gene lists (2021 update). Nucleic Acids Res 50:W216–W221
9. Subramanian A, Tamayo P, Mootha VK, et al (2005) Gene set enrichment analysis: A knowledge-based approach for interpreting genome-wide expression profiles. Proc Natl Acad Sci U S A. https://doi.org/10.1073/pnas.0506580102
10. Daňhelovská T, Zdražilová L, Štufková H, Vanišová M, Volfová N, Křížová J, Kuda O, Sládková J, Tesařová M (2021) Knock-out of acbd3 leads to dispersed golgi structure, but unaffected mitochondrial functions in hek293 and hela cells. Int J Mol Sci 22:7270
11. Pejznochova M, Tesarova M, Hansikova H, Magner M, Honzik T, Vinsova K, Hajkova Z, Havlickova V, Zeman J (2010) Mitochondrial DNA content and expression of genes involved in mtDNA transcription, regulation and maintenance during human fetal development. Mitochondrion 10:321–329
12. Stiburek L, Vesela K, Hansikova H, Pecina P, Tesarova M, Cerna L, Houstek J, Zeman J (2005) Tissue-specific cytochrome c oxidase assembly defects due to mutations in SCO2 and SURF1. Biochem J 392:625–632
13. Rodinova M, Krizova J, Stufkova H, et al (2019) Deterioration of mitochondrial bioenergetics and ultrastructure impairment in skeletal muscle of a transgenic minipig model in the early stages of Huntington’s disease. DMM Dis Model Mech. https://doi.org/10.1242/dmm.038737
14. Koralkova P, Belickova M, Kundrat D, et al (2021) Low Plasma Citrate Levels and Specific Transcriptional Signatures Associated with Quiescence of CD34 + Progenitors Predict Azacitidine Therapy Failure in MDS/AML Patients. Cancers 2021, Vol 13, Page 2161 13:2161
